# Inoperable malignant bowel obstruction: palliative interventions outcomes – mixed-methods systematic review

**DOI:** 10.1136/bmjspcare-2021-003492

**Published:** 2022-07-19

**Authors:** Michael Patterson, Sarah Greenley, Yangmyung Ma, Alex Bullock, Jordan Curry, Jacquelyn Smithson, Michael Lind, Miriam J Johnson

**Affiliations:** 1 Wolfson Palliative Care Research Centre, Hull York Medical School, University of Hull, Hull, UK; 2 Institute of Clinical and Applied Health Research, Hull York Medical School, Hull, UK; 3 Department of Plastic Surgery, University Hospitals of North Midlands NHS Trust, Stoke-on-Trent, UK; 4 Gastrointestinal and Liver services, Hull University Teaching Hospitals NHS Trust, Hull, UK; 5 Hull York Medical School, University of Hull, Hull, UK

**Keywords:** Intestinal obstruction, Gastrointestinal (lower), Genitourinary, Quality of life, Gastrointestinal (upper)

## Abstract

**Background:**

Parenteral nutrition (PN) and palliative venting gastrostomies (PVG) are two interventions used clinically to manage inoperable malignant bowel obstruction (MBO); however, little is known about their role in clinical and quality-of-life outcomes to inform clinical decision making.

**Aim:**

To examine the impact of PN and PVG on clinical and quality-of-life outcomes in inoperable MBO.

**Design:**

A mixed-methods systematic review and narrative synthesis.

**Data sources:**

The following databases were searched (from inception to 29 April 2021): MEDLINE, Embase, Cochrane Central Register of Controlled Trials, Web of Science, CINAHL, Bielefeld Academic Search Engine, Health Technology Assessment and CareSearch for qualitative or quantitative studies of MBO, and PN or PVG. Titles, abstracts and papers were independently screened and quality appraised.

**Results:**

A total of 47 studies representing 3538 participants were included. Current evidence cannot tell us whether these interventions improve MBO survival, but this was a firm belief by patients and clinicians informing their decision. Both interventions appear to allow patients valuable time at home. PVG provides relief from nausea and vomiting. Both interventions improve quality of life but not without significant burdens. Nutritional and performance status may be maintained or improved with PN.

**Conclusion:**

PN and PVG seem to allow valuable time at home. We found no conclusive evidence to show either intervention prolonged survival, due to the lack of randomised controlled trials that have to date not been performed due to concerns about equipoise. Well-designed studies regarding survival for both interventions are needed.

**PROSPERO registration number:**

CRD42020164170.

**WHAT IS ALREADY KNOWN ON THIS TOPIC**

- Clinical decision making in malignant bowel obstruction is complex, with a range of options available to the clinician.
- There are currently no national agreed guidelines to inform clinical decision making regarding malignant bowel obstruction management.

**WHAT THIS STUDY ADDS**

- Gastrostomy appears to be an effective intervention providing symptoms relief for patients with malignant bowel obstruction, allowing patients to spend time out of hospital and appears to improve quality of life for most.
- Parenteral nutrition plays a vital role in managing malignant bowel obstruction, allowing patients valuable time at home, and appears to improve quality of life for most but with associated burdens.

**HOW THIS STUDY MIGHT AFFECT RESEARCH, PRACTICE AND/OR POLICY**

- Parenteral nutrition and gastrostomy seem to support patients’ valuable time at home.
- Given the burdens associated with both interventions, healthcare professionals must present an honest and realistic account of the benefits and challenges of the treatment options.

## Introduction

Malignant bowel obstruction (MBO) is a serious complication of cancer, affecting an estimated 3%–15% of patients with cancer globally,^1^ and most common with primary cancers of gynaecological and gastrointestinal origin (50% and 28%, respectively).^1^ People with MBO describe distressing abdominal pain and distension, nausea and vomiting, inability to eat and drink with a consequential reduction in quality of life (QoL), nutritional and performance status.^2–5^

Surgery provides the best option for longer-term survival.^6–8^ However, surgery is often contraindicated due to ascites, peritoneal carcinomatosis, multiple sites of obstruction, and poor functional and nutritional status.^6^ Those with inoperable MBO (IMBO) are managed medically^9^ with analgesics, corticosteroids, antiemetics and antisecretory agents. Those with IMBO may also be managed with parenteral nutrition (PN), gut decompression (eg, palliative venting gastrostomy (PVG), nasogastric tube drainage) or stenting.^2 10^ Clinical decision making is challenging, with only low-level evidence to guide clinicians in day-to-day decision making with no nationally agreed recommendations leading to wide variation between clinical centres.^6 11 12^ Management choices are typically based on clinicians' individual clinical experience or patients’ goals (if explored).^6^

The use of PN in advanced cancer is receiving growing attention with the publication of systematic reviews^13–16^; two focused on MBO solely,^13 15^ two focused on advanced cancer, however, most included papers had a large proportion of participants with MBO.^14 16^ These reviews focused mostly on survival and rarely evaluated other important outcomes such as QoL and health resource utilisation.

There is only one systematic review exploring the use of PVG for MBO with regard to safety and efficacy for symptom relief^17^ but again, this did not address impact on QoL or health service utilisation.

We aim to synthesise systematically the current evidence on the use of PVG and PN in MBO, investigating how they affect: survival, health-related QoL, symptoms, health service utilisation, physical function and nutritional status. We included PVG for gut decompression or treatment with PN as destination treatment, with a comparator (if available) of no decompressive support or no PN support.

## Methods

The study is reported per the Preferred Reporting Items for Systematic Reviews and Meta-Analyses (PRISMA) guidelines.^18^

### Search strategy

The following databases were searched (from database inception to 2 March 2020): MEDLINE and Embase via OVID, CENTRAL via The Cochrane Library, Web of Science Core Collection, CINAHL Complete via EBSCOhost, Bielefeld Academic Search Engine (BASE) and CareSearch (see online supplemental file 1) for qualitative or quantitative studies of MBO, and PN and/or PVG, with no language limits.

10.1136/bmjspcare-2021-003492.supp1 Supplementary data

We searched for any currently recruiting trials in ClinicalTrials.gov (http://clinicaltrials.gov/), EU Clinical Trials Register (https://www.clinicaltrialsregister.eu/) and in the WHO International Clinical Trials Registry Platform (ICTRP) search portal (http://apps.who.int/trialsearch/).

The search was updated on 29 April 2021 using the search and screening strategy fully outlined in this paper from the 2 March 2020 to the 29 April 2021. The numbers of articles retrieved from each database and the two searches can be seen in online supplemental file 1.

Forward and backward citation searching of all included studies and relevant systematic reviews was completed: we examined the reference lists of included studies and identified articles citing included studies in Web of Science.

### Inclusion and exclusion criteria

Study eligibility criteria are detailed in table 1.

**Table 1:** Inclusion and exclusion criteria for identifying relevant studies via search strategies

**Inclusion criteria**	**Exclusion criteria**
PN	
People over 16 years of age with inoperable MBO. Receiving PN via a central venous catheter as destination palliative treatment.	Treatment with curative intent. Receiving PN through a peripheral vein. Receiving only intravenous fluids. Receiving enteral feeding alongside PN not deemed for quality of life. Patients were <16 years old. PN was administered preoperatively, peri-operatively or postoperatively to assess complications related to surgery.
PVG	
People over 16 years of age with inoperable MBO. Receiving gut decompression via a PVG tube or nasogastric tube as destination palliative treatment. Studies that include patients with both benign and malignant diseases if the results were reported separately for each group.	Treatment with curative intent. Patients were <16 years old. PVG insertion for decompression in non-malignant disease.

MBO, malignant bowel obstruction; PN, parenteral nutrition; PVG, palliative venting gastrostomies.

### Study selection

All titles and abstracts retrieved by electronic searching were downloaded to an Endnote 20 library, and duplicates removed according to a published protocol.^19^ The remaining articles were uploaded to the online citation-screening tool Covidence.^20^ Studies were dual screened independently (MP, YM) based on title and abstract for eligibility. Full-text articles were also retrieved in the case of uncertainty. Full texts were reviewed by two authors (MP, YM, AB and JC). Any disagreements were resolved through consensus.

### Data extraction

Data were extracted using a piloted and modified bespoke form. MP extracted data from all studies, and YM and AB each from a random 25%.

### Quality assessment

Randomised controlled trials (RCTs) were assessed against the Risk of Bias 2.0 tool.^21^ All cohort studies were appraised against the Critical Appraisal Skills Programme cohort checklist tool^22^ items 1–10. All qualitative studies were evaluated against the Critical Appraisal Skills Programme qualitative checklist tool^23^ items 1–10 (see online supplemental file 2).

10.1136/bmjspcare-2021-003492.supp2 Supplementary data

### Analysis

The Joanna Briggs Institute convergent segregated approach to synthesis and integration was followed; this consists of conducting separate quantitative synthesis and qualitative synthesis, followed by integrating evidence derived from both.^24^

For the qualitative synthesis, the direct quotation data were synthesised by MP and AB using thematic synthesis.^25 26^ This allowed the context of each study to be considered while aiming to produce a generalisable synthesis.^25^ Participant quotes and the authors’ interpretations were used. The below analysis was conducted on paper with the final analysis broken down into quotes, codes, subthemes and themes (online supplemental file 3). Three stages were conducted: (1) initial data coded regarding experiences of PN and PVG (MP, AB); (2) descriptive themes generated, with codes grouped into categories (MP, AB) and (3) analytical themes generated both inductively and deductively, with the investigators (AB, MP) generating themes independently, then through discussion with a third investigator (MJJ). A decision was made to combine the findings from the PN and PVG literature as the themes arising were common throughout.

10.1136/bmjspcare-2021-003492.supp3 Supplementary data

For the quantitative synthesis, due to significant heterogeneity, a narrative summary only was completed.

## Results

**Figure 1:**
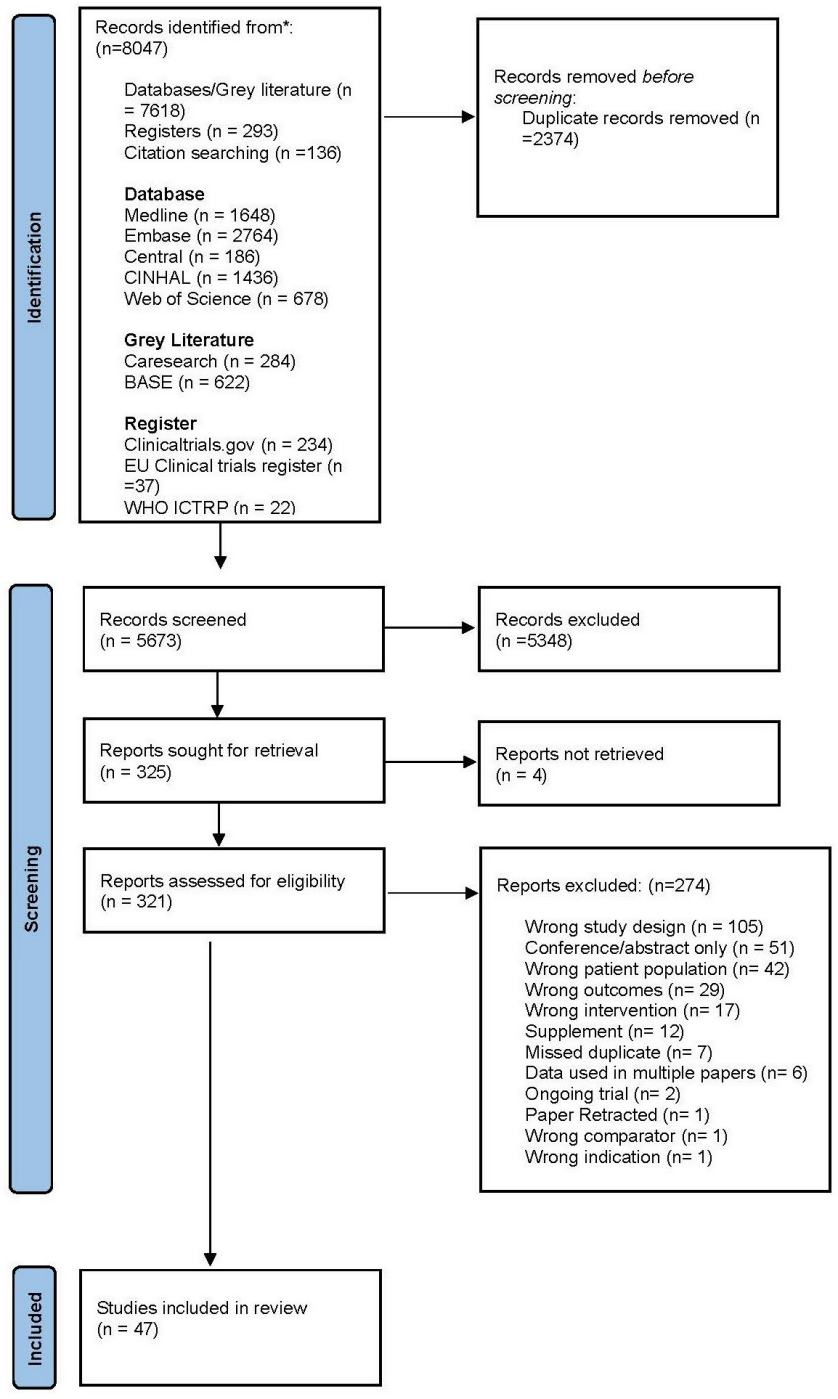
Identification of studies via databases and registers.

The search returned 5673 unique articles after deduplication. From this, 47 studies, representing 3538 participants, including 30 participants from four qualitative studies, published between 1992 and 2021, were included (see PRISMA flow chart, figure 1)^18^

### Study characteristics: quantitative

Online supplemental file 4 provides summary descriptions of the included quantitative studies. There were 6 prospective,^27–32^ 35 retrospective cohort studies,^7 8 33–67^ 1 mixed-method study^68^ and 2 RCTs.^69 70^ Sample sizes ranged from 7 to 629. Studies were globally represented; 1 study from Australasia,^50^ 5 from Asia,^31 40 60 69 70^ 17 from Europe^27–30 32 35 39 42 44–47 54 64 67 68^ and 21 from North America.^7 8 33 34 36–38 43 49 52 53 55–59 61–63 65 66^

10.1136/bmjspcare-2021-003492.supp4 Supplementary data

### Parenteral nutrition

#### Participants

Twenty-one studies were included, with 1884 participants (age ranged from 22 to 88 years; females 61%). The underlying primary malignancy was the gastrointestinal tract in just over half (53%) of patients, gynaecological in a quarter (24%) of patients (accounting for the female predominance) and other sites in a further quarter (26%). MBO was reported in 100% of patients in 14 studies^29 33–35 37–40 44 45 47 68 70^ and between 72% and 90% in the other 7 studies^30–32 42 43 46 48^; overall, 94% of included patients (see online supplemental file 3).

#### Survival

All PN studies reported on overall survival. However, the definition of length of survival was inconsistent, with seven definitions for survival given, with no definition in one study,^31^ reflecting different study populations (see online supplemental file 4). The possibility of combining quantitative data for a meta-analysis regarding survival was explored, but due to significant heterogeneity, a narrative summary only was completed.^71^

Seventeen studies reported median survivals ranging between 13 and 143 days (range: 2–2111 days).^29–34 36–39 42 43 45–47 68 70^ Seven studies reported mean survivals between 30 and 198 days (range: for 1–1715 days).^35 40 40 41 44 44 48^

One retrospective cohort study found that those receiving PN in addition to anticancer treatment (chemotherapy) had a longer median survival (89 vs 71 days, (p=0.031)).^33^ A prospective^32^ and another retrospective cohort study^39^ found that those receiving PN in addition to anticancer treatment had longer 3-month and 6-month survivals (p<0.00001.^39^

Only two retrospective cohort studies compared survival in those receiving PN compared with those who did not as a sole intervention. Those receiving PN lived longer (323 vs 91 days, p=0.0021^45^; 72 vs 41 days, p=0.01.^36^ Though for one study^45^ this improved survival compared those who received PN to those who did not despite being assessed retrospectively as eligible by the study team.

Two retrospective cohort studies^42 48^ and one prospective cohort study^31^ showed a positive association between performance status and survival; a Karnofsky performance status >50 at baseline was associated with longer survival.

A further retrospective cohort study^39^ found similar results using the Eastern Cooperative Oncology Group performance status, rated from 0, fully active, to 5, dead. They found baseline performance status impacted on survival (0=median 680 (range 543–1393); 1=median 174 (65–748); 2=median 61.5 (25–399); 3=median 26 (16–64) days).

### Health-related QoL

A prospective cohort study^32^ found an improvement over 3 months for global QoL, physical, role and emotional functioning, as well as appetite loss and fatigue. An additional retrospective cohort study^30^ reported physical, psychological, and activity assessments; roughly half deteriorated and 40% improved—with a small percentage showing no change using the Rotterdam symptom checklist. In contrast, only a quarter of patients showed a worsening of the well-being assessment.

A retrospective cohort study^43^ used non-validated measures but saw a statistically significant improvement in gastrointestinal discomfort, nausea, vomiting, fatigue level, morale and social interactions during home PN use as compared with prehome PN status (p=0.05). Those with a prehome PN and Karnofsky >40 had greater improvement in QoL than those with worse performance status (see online supplemental file 3).

### Performance status

Only three studies reported the impact of PN on performance status (table 2). A prospective cohort study^30^ and a retrospective cohort study^43^ found performance status was maintained. A further retrospective cohort study found an improvement (most marked in those living longer).^47^ However, patients with a KPS <30 were excluded from these studies.

**Table 2:** Parenteral nutrition and performance status

Study	Performance status metric	Numbers performance status measured	Time point	Score
Bozzetti *et al* 30 2002	KPS	69	Baseline	Median 60
		69	‘Until 3 months prior to death’	‘stable’
King *et al* 43 1993	KPS	72	Baseline	48
		72	‘During home parenteral nutrition’	47
Santarpia *et al* 47 2006	KPS	In 64 patients who survived longer than 60 days		Mean
		64	Baseline	≤40 12 ≥50 52
		64	1 month	≤40 10 ≥50 54
		In 39 patients who survived longer than 90 days		Mean
		39	Baseline	≤40 5 ≥50 34
		39	1 month	≤40 4 ≥50 35
Ruggeri *et al* 46 2020	Karnofsky Performance Status Scale	Precachexia		Mean
		249	Baseline	56
		249	1 month	58
		Cachexia		Mean
		478	Baseline	52
		478	1 month	53
		Refractory cachexia		Mean
		242	Baseline	49
		242	1 month	49

### Nutritional status

One prospective cohort study^30^ reported home PN maintained the same nutritional status assessed at the start of treatment until death. Four retrospective cohort studies^42 43 45 47^ found an improvement in body weight of those on home PN (improvement greater in those living longer). Interestingly, one^45^ found the opposite was true; those who were not referred for PN had decreases in weight over time. Findings summarised in table 3.

**Table 3:** Parenteral nutrition (PN) and nutritional status

Study	Numbers performance status measured	Time point	Weight (kg)	
Bozzetti *et al* 30 2002	69	Baseline	Median- 52.5 (range 35.5–77.5)	
	69	Time of death	Median 54.0 (range 36–78)	
Keane *et al* 42 2018	71	Starting PN	55.3±12.3	
	37	Outpatient clinic 0–3 months	54.5±9	
	19	Outpatient clinic 3–6 months	58.6±11.3	
King *et al* 43 1993	61	Pre-home parenteral nutrition	Mean (±SD) 54.5±13.7	
	55	1 week	Mean (±SD) 56.4±12.8	
	50	1 month	Mean (±SD) 57.2±12.4	
	18	3 months	Mean (±SD) 57.7±11.2	
	9	6 months	Mean (±SD) 59.8±11.7	
	7	1 year	Mean (±SD) 57.4±8.3	
Patel^45 77^			Referred for PN	Not referred for PN
	105	At obstructive episode	Median (range) 53.9 (41.8–89) n=47	Median (range) 57 (38-100) n=58
	60	At 0–3 months follow-up	Median (range) 54 (39.9–82.8) n=31	Median (range) 55.0 (41.8–89) n=29
	39	At 4–6 months follow-up	Median (range) 55.7 (38.7–85.4) n=22	Median (range) 55.8 (41.8–89) n=17
Santarpia *et al* 47 2006		In 64 patients who survived longer than 60 days		
	64	Baseline	Mean (±SD) 51.7±10.3	
	64	1 month	Mean (±SD) 53.2±10.3	
		In 39 patients who survived longer than 90 days		
	39	Baseline	Mean (±SD) 50.5±10.2	
	39	1 month	Mean (±SD) 52.0±10.1	

### Symptoms

No studies reported on symptoms unless reported in QoL data.

### Health service utilisation

The available health service utilisation data came from seven retrospective cohort studies (online supplemental file 5).^34 35 37–39 43 45^

10.1136/bmjspcare-2021-003492.supp5 Supplementary data

Readmission rates were variable with low medians between 0 and 2 but a wide range of 0–13^37 39 43 45^ Reasons for readmissions were limited and time frames were lacking. One study^43^ reported 11/124 (9%) hospitalisations were for home PN-related complications, the others being for cancer therapy or disease complications. Two further studies reported on readmissions, one^38^ reporting 1/9 (11%) had five readmissions. The second^34^ reported 3/18 (17%) were readmitted to evaluate possible home PN-related complications.

Intensive care unit (ICU) admissions were reported in two studies and varied widely (from a median of 0^45^ to 23/82 (28.1%)^37^).

Median length of stay ranged from 10.1 to 26.5 days.^35 37 45^ With one^45^ study reporting a greater length of stay in those referred for PN than those who were not (28 vs 9 days, p=0.0001).

### Place of death

Three retrospective cohort studies reported on place of death (online supplemental file 5).^36 42 45^ Most patients died in their home or hospice (range 68%–81.3%) reported across the three studies.

### Palliative venting gastrostomy

#### Participants

Twenty-three studies were included for quantitative analysis, with 1657 participants (age ranged from 20 to 95 years; females 78%) (online supplemental file 4). The underlying primary malignancy was gynaecological in 57% of patients (accounting for the female predominance), the gastrointestinal tract in 37% of patients and other sites in 6%. All participants in all studies had MBO.

#### Survival

All studies reported the overall survival of participants with PVG, again, defining survival from different points, or not defined in one^8^ (see online supplemental file 4). Survival was however heavily confounded by the varying use of PN post-PVG.

Thirteen studies reported median survivals between 13 and 63 days, range from 1 to 1226 days.^7 27 49 50 53 54 56 59–63 67^ Five studies reported mean survivals between 53 and 135 days, range from 5 to 2772 days.^28 52 57 58 65^ Two studies reported ‘average survival’ between 83.7 and 147 days, range 20–364 days.^64 66^

One study reported percentage alive at 30 days, 1 year and 3 years, of 54.8%, 11.43% and 9.5%, respectively.^55^ One study stated survival of 50 days for the PVG group and 86 days for the nasogastric tube group without further qualification of the measure.^69^

#### Quality of life

An RCT^69^ found higher QoL scores for PVG versus nasogastric tube for both EuroQl-5D (mean—7.132 (4.543–9.702) vs 3.663 (0.464–6.862)) and ShortForm-8 scores (mean—420.1 (282.6–557.6) vs 199.4 (22.2–376.6)).

A retrospective cohort study^67^ had 25 completed symptoms Distress Scale scores. Sixteen (64 %) improved (41 vs 32.6, pre-PVG and post-PVG median scores, respectively, p≤0.01), two (8 %) showed the same scores as at baseline, and seven (28 %) had non-significant worsening (30.85 vs 36.14, p=0.18) of QoL (see online supplemental file 4).

#### Performance status

No studies reported on performance status.

#### Nutritional status

No studies reported on nutritional status.

#### Symptoms

Two prospective cohort^27 28^ and thirteen retrospective cohort studies^49 50 52 57–60 62–67^ reported a reduction in nausea and vomiting in 657/750 (88%) participants. A further retrospective study showed that PVG significantly reduced the daily frequency of vomiting to 18% of the initial value, and a reduced probability of nausea to 50% (both p<0.001).^54^

One prospective cohort^28^ and seven retrospective cohort studies^57 59 62–65 67^ reported whether participants were able to resume an oral diet, either liquid or soft diet, following insertion of PVG. Where noted, ability to tolerate some sort of diet was achieved in 353/432 (82%). A retrospective cohort study^50^ reported the ability to resume some oral intake was usually viewed by patients and families positively (see online supplemental file 3).

#### Health service utilisation

See online supplemental file 5

Hospital readmission rates varied from 11/96 (11.4%)^58^ to 4/7 (47%).^65^ Reasons for readmission were reported; PVG-related events between 4/96 (4%)^58^ and 48/115 (42%),^53^ recurrent ‘average’ length of time spent at home prior to readmission was 21.7 days (range 5–60 days)^59^ to 126 days (range 7–467 days).^65^

Median length of stay varied from 6 to 23 days (range 1–60). An additional retrospective cohort study reported median length of stay prior to placement of PVG of 6 days (range 1–27).^64^

Twenty/51 (39%)^50^ to 83% (20/24)^64^ of patients with PVG were discharged home. Hospice enrolment rates varied from 5/53 (9.4%)^63^ to 95/117 (81%).^56^ A further retrospective cohort study^67^ reported 116/158) (81.6% of patients discharged, though discharge location unknown).

The largest study^7^ included in the review was a retrospective cohort study of 3583 people. They found PVG use was associated with lower intensity hospital service utilisation (higher hospice enrolment, fewer readmissions, ICU admissions and hospital deaths) at the end of life, compared with medical management or surgery to manage MBO. While this was a retrospective cohort study the authors used regression models to adjust for patient and hospital covariates to account for confounders.

#### Place of death

Death in hospital was wide ranging from 2/53 (4%) to 4/7 (57%).^65^ There were few data about death outside of hospital (online supplemental file 5). Of the data available proportions of those dying at home ranged from 6/51 (12%)^50^ to 3/7 (43%).^65^ Another retrospective cohort study^62^ reported on home or hospice care with 75 of 88 (85%) patients dying at home or under hospice care. Unfortunately for most studies the place of death for most patients is unknown.

A retrospective cohort study^54^ reported deaths for their full cohort: hospital n=46 (61%), home n=23 (30%), and inpatient hospice n=6 (9%). A further study^54^ reported discharge disposition of their full cohort, presumed to be place of death: home n=22 (40%), rehabilitation n=7 (15%) and hospice n=25 (45%).

#### Quality of included studies

The general quality of the observational studies was poor, with the majority being retrospective studies without a comparator (see online supplemental file 2). The studies did not sufficiently address confounding variables, such as performance status, and biases such as no randomisation to treatment groups, and no blinding of participants or healthcare professionals. Likewise, the risk of bias in the RCTs was high and none compared either PN or PVG with usual care alone. The quality of the qualitative studies was of higher quality, though generalisability was inherently limited by its narrow focus; this not being an aim of qualitative research.

## Qualitative synthesis

### Parenteral nutrition

Three studies^68 72 73^ were included; all reported findings from 57 interviews from the same study group: 20 women with ovarian cancer, mean age 67 (±SD 7.5), and 13 family caregivers.

### Palliative venting gastrostomies

One study^74^ was included. The study included 11 participants (10/11 women; 7 with gynaecological cancer and 4 with colorectal cancer). Twelve interviews were conducted: 11 initial face-to-face interviews and 1 telephone reinterview.

### Interview findings

All quotes are from patients unless otherwise highlighted and are shown in online supplemental file 3.

Two key themes emerged: (1) A stark decision: do or die; (2) Hope versus reality of the intervention.

### A stark decision: do or die

Patients and carers felt there was no good alternative to PN. They viewed the choice as between life (PN) or death (starve).

It’s either die with food or (home PN) for the rest of your days and I’d sooner live and be on (PN)^68^

Well, to me it was a no option thing. I don’t think they could have done anything else, bar starve me… if that’s what’s keeping me alive, it’s what I have to have isn’t it. So I don’t think (there was) a decision as such, if there was no other… if I can’t eat, it will be next best thing(PN)^74^

Whether this belief was a result of over-optimistic emphasis from clinicians on possible survival benefits (given the lack of level 1a evidence regarding survival) or received in this manner because of the serious nature of the situation was not clear. Whichever, with such stark alternatives, most were trusting of their clinical team and felt they had little choice but to agree with a decision already made.

Certainly yes, I mean what’s the alternative…you just have to go with what the doctors recommend, I think. (PVG)^74^

### Hopes versus realities of the intervention

The interventions themselves brought benefits in perceived quantity and QoL; a view held by both patients and carers.

Spending time with family when you get to, like, my stage, is the most important for everybody (PN)^68^

It’s keeping her alive really. That’s the big advantage. (Husband). (PN)^68^

For some the benefits were the control of symptoms or improved function.

Well they explained that it would be helpful for the sickness…stopping the sickness, which it did. I was so grateful for that because it was just projectile all the time. (PVG)^74^

it’s given me, yes, more energy (PN)^68^

However, both interventions brought their own burdens. For both the patients and the carers these burdens were more than they had expected.

initially when this was being discussed with us … I thought it was probably less medical than what it is (Daughter). (PN)^68^

It wasn’t as easy as it was made out to be” (PN)^68^

This underestimation of the impact included the procedures involved, especially if written information was not given prior to the intervention.

when I got down to radiology, Dr X (Consultant IR) came and explained it all to me and I was even more anxious then because I sort of then understood what was happening… (PVG)^74^

The physical burden of the intervention on both patient and carer was considerable, with many participants managing both PN and PVG together.

(are you able to walk up and down the stairs?) …not when carrying my bags (referring to her PVG, PN and syringe pump), but X (partner) carries those either behind or in front of me. (PN)^68^

My husband has been in a lot of discomfort, it has been leaking all the time, he’s being changed numerous times a day, the beds have to be changed and now his skin is all sore. (PVG)^74^

Alongside the physical burden, an emotional burden was expressed by patients, which was often echoed by carers.

It would be wonderful if I could have even 5 hours sleep without a break (PN)^68^

You can smell it though, even if it’s not leaking. I feel like…it smells like sewage, it’s not faecal, it’s worse than that, it’s a sewage smell and I feel like I can smell it all the time and anyone who is anywhere near me can smell it. It is making me quite paranoid; I am constantly asking my husband if he can smell it…I don’t get embarrassed too easily, but I do find that quite difficult to deal with) (PVG)^74^

The emotional burden was apparent, particularly when the duration of care went on, and hypervigilance and sleeplessness aggravating the distress.

I’m awake most of the night listening for her, but she tells me not to help her(Husband) (PN)^68^“

What you sign on for when you get married (Husband) (at the end of the second interview, he reported feeling like a ‘prisoner’) (PN)^68^

Although stoicism and resilience and adaptation by many to a new normal was apparent, the sense of what had been lost was felt keenly.

when I go in the shower and everything, I can … take both tubes off, and I’m a different person (PN)^68^

It just becomes a way of life really, you know what I mean, this is how your day goes and this is what it is. A nurse comes and takes it off in a morning and then a nurse comes at night and puts it back on (PN)^68^

## Integration of quantitative and qualitative evidence

Each outcome of interest was determined to be in concordance, dissonance or silent from the quantitative evidence or qualitative studies using the convergent segregated approach to synthesis and integration.^24^ This methodology allows exploration of the results of findings from the quantitative and qualitative synthesis to examine if there is agreement (concordance), disagreement (dissonance), or have no relationship or not mentioned (silence).^24^

One of the primary outcomes was survival. There was dissonance between the quantitative and qualitative data. The qualitative data showed that participants believed that the decision represented ‘do or die’, but this was not substantiated by the quantitative data as the quality of the evidence was such that we could not demonstrate a survival advantage with either parenteral nutrition or gastrostomy to allow clinicians to present prolonged survival with any certainty. This dissonance is likely due to patients perceptions, patients viewed that clinicians made the decision for them, often out of clinical necessity.^72^ This decision-making process has been echoed by numerous studies^75 76^ were patients feel there is no decision to be made if there was only one treatment option. In this case patients make the choice to live and then by necessity accept whatever they perceive will facilitate this, in this case VPG or PN. A further potential for this dissonance could be patients’ misconceptions about the benefits of noncurative cancer treatment, highlighted by numerous studies,^77–79^ these misconceptions can be influenced by coping mechanisms such as hope and emotional factors that drive decision making.^77 80 81^

Both sources of data were concordant regarding net improvement in QoL despite significant burdens for those with a gastrostomy. No participant regretted insertion and would recommend gastrostomy to others. The QoL parenteral nutrition quantitative data are less clear; for some participants there was obvious improvement, but not for all. However, this was concordant with the qualitative data with a gain for some while others reported significant burdens. However, it appears that participants were willing to live with the burdens because they believed this would bring survival benefit.

For parenteral nutrition, there was silence in both data sources for symptoms, if it is captured at all it is seen as part of QoL data, such as physical function and fatigue. With gastrostomy data findings were concordant: high symptom relief reported quantitatively and echoed in the qualitative data.

With parenteral nutrition there are few data regarding nutritional or performance status. The data available point to a maintenance of performance status for most, with an improvement in some. Nutritional status seems to be improved with parenteral nutrition. In the qualitative data there is some mention of improvements in energy levels or self-reported weight gain. For gastrostomy there is silence on both accounts.

For both health service utilisation and place of death there is silence for both interventions in the qualitative data. This is due to the focus of the research questions which did not explore the impact on place of care and provides questions for future research.

## Discussion

We provide the first mixed-methods systematic review and synthesis of PN and PVG in MBO, investigating a range of patient-relevant outcomes. Forty-seven papers, representing 3538 participants, were included.

Both interventions improved QoL, especially with PVG, and on balance for PN, where the benefits outweighed the burdens of the intervention in the context of a perceived threat of death as an alternative. No patients regretted the decision to have a PVG.

We could not determine whether PN prolonged survival, this systematic review found no level 1a (evidence from RCTs) with regard to survival or level 2a evidence (well-designed observational studies which address key confounders), this review echoes that of the Cochrane review.^14^ The lack of RCT evidence is discussed below. However, it is notable that for a significant proportion of patients receiving PN, there appears to be a survival advantage of months. This suggests a PN-related survival advantage for particular subgroups such as those with earlier-stage disease unable to tolerate oral and enteral nutrition when compared with starvation.

Two studies compared survival with PN to no PN, but the observational study designs were unable to account for significant confounding baseline variables, such as stage of disease or performance status. The only RCT for PN is one phase-2 trial, comparing IV hydration to PN, with poor recruitment resulting in insufficient power,^70^ the median survival of the PN group was 13 days, highlighting these patients were dying from advanced stage of the tumour not from starvation. It is argued that only if the patients are expected to die from starvation before they die from advanced cancer, there is a rationale for a trial of PN. The need for more definitive data regarding survival is clear, as our qualitative data shows that a belief in increased survival is the primary motivator for patients consenting to treatment.

A key clinical challenge is identifying patients who are likely to survive for long enough to benefit from PN. Existing guidelines^82–84^ suggest those with an expected prognosis of 2–3 months or greater, and those with a higher performance status may benefit most from PN with regard to survival. However, our review shows that we do not know whether apparent improvements in survival are merely a feature of baseline performance status (those with better performance status are also those most likely to get PN).

Our median survival ranges are consistent with other work.^15 16^ However, included studies used seven different definitions for measuring survival, which, alongside the skewed survival data and various methods for reporting averages, made it inappropriate to combine the study results. Cochrane authors haven taken the same view, finding the same problem.^11 13^

We have no level 1a evidence, or robust evidence from large observational studies which account for confounding variables (especially stage and amount of disease) and documented harms from PN, although again with lack of clarity how these affect any net benefit. Therefore there is ethical equipoise^85^ with regards to an RCT—at least in those who do not have stage 1 disease, or a single site of obstruction from localised disease. With unproven effectiveness and documented harms from PN, this should be of concern to clinicians and patients.

However, given the strong belief (clinicians and patients) that death would be due to due to starvation in most, if not all, cases, we recognise that an RCT would be very difficult to carry out due to reluctance of both clinicians and patients regarding randomisation. The unsuccessful phase 2 RCT we include^70^ illustrates this challenge, but the authors do not describe their process of consent, or how they may or may not have addressed the issue of equipoise during recruitment and consent. A successful RCT would need careful inclusion criteria (the population where there is most doubt) and extensive education to both clinical site staff and potential participants with regard to ethical equipoise. A well-designed feasibility RCT across several large oncology and intestinal failure centres which included appropriate and well delivered education during recruitment would be needed to assess whether or not a RCT would indeed not be possible.

Survival and PVG use data were largely confounded as many of those who received a PVG also received PN. Unlike PN there is a less strong plausible physiological rationale for a survival benefit, other than the potential of reduced mortality and morbidity through reducing the risk of aspiration. Nonetheless, as with the PN data, patients perceived PVG to provide a survival benefit and again this was a key determinant for agreeing to PVG placement.

Symptoms were improved by PVG but not measured or discussed for PN. Burdens (to patients and carers) were an issue for both interventions, with the reality often at odds with the expected experience, with some not being prepared for the impact of both the process of having the intervention, and of living with it.

Performance and nutritional status appear to be maintained, or improved, by PN. Our review demonstrates a potential relationship between performance status and anticancer treatments which may increase survival in this situation. Nonetheless, for most outcomes data were sparse and drawn from low quality evidence. A potential area of further investigation is whether PN improves performance status enough to allow further anticancer treatment in those previously deemed unsuitable.

For both interventions, health service utilisation and place of death data were variable, and the impact on these outcomes is unclear. Health service utilisation data were descriptive, highlighting that around 80% of patients die at home or in hospice care. Readmissions overall are low, but for a subgroup are many, likely reflective on the varying disease stages and performance statuses. Both interventions appear to allow patients to spend time out of hospital and valuable time at home. One of the largest studies to investigate health service utilisation within MBO^7^ concluded that PVG is associated with fewer readmissions and lower intensity healthcare utilisation at the end of life, compared with medical management or surgery.

A place of death outside of hospital could be a motivating factor for choosing these interventions. This was demonstrated in the PN qualitative literature, which highlighted that a key benefit of the intervention was allowing time at home with loved ones. Previous research has also emphasised for those with advanced cancer home care is the most common preferred place of death, with inpatient hospice care as second preference^86^

Of note, no studies compared PN or PVG with medical or surgical management alone. Two RCTs were included in this review, one of which compared PVG to nasogastric tube,^69^ showing greater symptom management and QoL for the PVG. This suggests that PVG placement needs to be considered earlier in the decision-making process to avoid repeated nasogastric tube insertions. The second^70^ comparing intravenous fluids to PN was only able to recruit 31 of a proposed 116 patients; many patients and families were ‘repulsed’ by the idea of the study due to their distresses regarding a patient starving to death if allocated to the control arm. The ethical considerations in this area are numerous, and centre on the randomisation of nutrition to patients who are unable to eat, particularly if studies aim to include a ‘no treatment arm’. This is an ongoing dilemma and barrier to MBO research^87^ and highlights the importance of accurate understanding and appropriate communication by professionals about the known benefits of interventions. This is evidenced by a paucity of well-designed prospective clinical trials.

### Implications for clinical practice and research

It appears PN plays a key role in the management of MBO in allowing patients valuable time at home. However, healthcare professionals need to be aware of the emotional and physical costs that patients and their carers will face. They must present an accurate picture when deciding on treatments. Further data on QoL and survival are necessary before more informed decisions regarding the usefulness of PN in palliative MBO can be made. Due to the feasibility challenges of undertaking RCTs with this intervention, the feasibility of randomisation should be identified before conducting a phase-3 RCT. If proven to be unfeasible, an alternative study design could be a quasi-RCT where patients with IMBO who would qualify for PN but choose not to be treated with PN act as the control group, but again this would be challenging to recruit to given the health beliefs regarding the benefits of PN. Further research in relation to the decision-making processes for PN is also required, and as patients view these decisions as clinician led, a greater understanding of clinicians’ decision making process is needed.

It appears that PVG is an effective intervention providing symptoms relief for patients with MBO, allows patients to spend time out of hospital and appears to improve QoL for most. Gastrostomies appear to be an underutilised intervention in clinical practice, and uptake of their use could be improved, though not without realistic information being provided to help patients make more informed decisions on their use. A direction for further research is regarding gastrostomies and patient QoL. As PVG appears to be underutilised, as with PN, a greater understanding of clinicians’ decision-making processes is required.

### Strengths and limitations

The use of a mixed-methods design is the main strength of this review, with both qualitative and quantitative studies being included in the analysis. This allows the triangulation of results and enables a richer insight into patients’ experiences of PN and PVG.

There are several limitations. First, due to varying definitions for outcomes, and study quality, a meta-analysis of extracted data was not possible. Second, the studies or components of studies were judged to be of variable quality and subject to varying risk of bias. Overall, the certainty of evidence was very low, derived mainly from observational studies without a comparator, and without robust adjustment for major confounders. Finally, with the qualitative data few papers were found, illustrating that this is currently under-researched, with PN data drawn from one cohort of women with ovarian cancer, and PVG data drawn from 11 patients, only 1 of which was male.

## Conclusion

PN and PVG may support patients’ valuable time at home.

PVG also provides symptom relief and better QoL, and participants would recommend the intervention to others. We found no high quality evidence to show either intervention prolonged survival, but this was a firm belief by patients and clinicians, providing the context for their decision making. Given the burdens associated with both, and that reality was different to expectations, healthcare professionals must present and honest and realistic account of the benefits and challenges of the treatment options. Well-designed studies should be done to address the knowledge gap regarding survival for both interventions and symptom benefits for PN. We need to identify patients most likely to benefit from PN or PVG.

## References

[1] Tuca A , Guell E , Martinez-Losada E , et al . Malignant bowel obstruction in advanced cancer patients: epidemiology, management, and factors influencing spontaneous resolution. Cancer Manag Res 2012;4:159–69. 10.2147/CMAR.S29297 22904637 PMC3421464

[2] Ripamonti CI , Easson AM , Gerdes H . Management of malignant bowel obstruction. Eur J Cancer 2008;44:1105–15. 10.1016/j.ejca.2008.02.028 18359221

[3] Caparica R , Amorim L , Amaral P , et al . Malignant bowel obstruction: effectiveness and safety of systemic chemotherapy. BMJ Support Palliat Care 2020. 10.1136/bmjspcare-2020-002656 [Epub ahead of print 17 Dec 2020]. 33334819

[4] Selby D , Wright F , Stilos K , et al . Room for improvement? A quality-of-life assessment in patients with malignant bowel obstruction. Palliat Med 2010;24:38–45. 10.1177/0269216309346544 19797338

[5] McCaffrey N , Asser T , Fazekas B , et al . Health-related quality of life in patients with inoperable malignant bowel obstruction: secondary outcome from a double-blind, parallel, placebo-controlled randomised trial of octreotide. BMC Cancer 2020;20:N.PAG-N.PAG. 10.1186/s12885-020-07549-y PMC7603764 33129304

[6] Bateni SB , Gingrich AA , Stewart SL , et al . Hospital utilization and disposition among patients with malignant bowel obstruction: a population-based comparison of surgical to medical management. BMC Cancer 2018;18:1166. 10.1186/s12885-018-5108-9 30477454 PMC6258444

[7] Lilley EJ , Scott JW , Goldberg JE . Survival, healthcare utilization, and end-of-life care among older adults with malignancy-associated bowel obstruction: comparative study of surgery, Venting gastrostomy, or medical management. Annals of Surgery 2018;267:692–9. 28151799 10.1097/SLA.0000000000002164 PMC7509894

[8] Merchant SJ , Brogly SB , Booth CM , et al . Management of cancer-associated intestinal obstruction in the final year of life. J Palliat Care 2020;35:84–92. 10.1177/0825859719861935 31307272

[9] Krouse RS . Malignant bowel obstruction. J Surg Oncol 2019;120:74–7. 10.1002/jso.25451 30908650

[10] Franke AJ , Iqbal A , Starr JS , et al . Management of malignant bowel obstruction associated with Gi cancers. J Oncol Pract 2017;13:426–34. 10.1200/JOP.2017.022210 28697317

[11] Cousins SE , Tempest E , Feuer DJ . Surgery for the resolution of symptoms in malignant bowel obstruction in advanced gynaecological and gastrointestinal cancer. Cochrane Database Syst Rev 2016:CD002764. 10.1002/14651858.CD002764.pub2 26727399 PMC7101053

[12] Bleicher J , Lambert LA , Scaife CL , et al . Current management of malignant bowel obstructions: a survey of acute care surgeons and surgical oncologists. Trauma Surg Acute Care Open 2021;6:e000755. 10.1136/tsaco-2021-000755 34222676 PMC8211049

[13] Sowerbutts AM , Lal S , Sremanakova J , et al . Home parenteral nutrition for people with inoperable malignant bowel obstruction. Cochrane Database Syst Rev 2018;8:CD012812. 10.1002/14651858.CD012812.pub2 30095168 PMC6513201

[14] Tobberup R , Thoresen L , Falkmer UG , et al . Effects of current parenteral nutrition treatment on health-related quality of life, physical function, nutritional status, survival and adverse events exclusively in patients with advanced cancer: a systematic literature review. Crit Rev Oncol Hematol 2019;139:96–107. 10.1016/j.critrevonc.2019.04.014 31150954

[15] Naghibi M , Smith TR , Elia M . A systematic review with meta-analysis of survival, quality of life and cost-effectiveness of home parenteral nutrition in patients with inoperable malignant bowel obstruction. Clinical Nutrition 2015;34:825–37. 10.1016/j.clnu.2014.09.010 25288565

[16] O’Hanlon FJ , Fragkos KC , Fini L , et al . Home parenteral nutrition in patients with advanced cancer: a systematic review and meta-analysis. Nutr Cancer 2021;73:943–55. 10.1080/01635581.2020.1784441 32586120

[17] Thampy S , Najran P , Mullan D , et al . Safety and efficacy of Venting gastrostomy in malignant bowel obstruction: a systematic review. J Palliat Care 2020;35:93–102. 10.1177/0825859719864915 31448682

[18] Page MJ , Moher D , Bossuyt PM , et al . PRISMA 2020 explanation and elaboration: updated guidance and exemplars for reporting systematic reviews. BMJ 2021;372:n160. 10.1136/bmj.n160 33781993 PMC8005925

[19] Bramer WM , Giustini D , de Jonge GB , et al . De-duplication of database search results for systematic reviews in endnote. J Med Libr Assoc 2016;104:240–3. 10.3163/1536-5050.104.3.014 27366130 PMC4915647

[20] Covidence . Covidence systematic review software Melbourne, Australia: veritas health innovation, 2021. Available: www.covidence.org [Accessed 29 Apr 2021].

[21] Sterne JAC , Savović J , Page MJ , et al . Rob 2: a revised tool for assessing risk of bias in randomised trials. BMJ 2019;366:l4898. 10.1136/bmj.l4898 31462531

[22] CASP . Cohort study checklist. In Critical Appraisal Skills Programme. Available: https://casp-uk.net/casp-tools-checklists/2018https://casp-uk.net/casp-tools-checklists/

[23] CASP . Casp qualitative studies checklist. Available: https://casp-uk.net/casp-tools-checklists/2018https://casp-uk.net/casp-tools-checklists/

[24] Stern C , Lizarondo L , Carrier J , et al . Methodological guidance for the conduct of mixed methods systematic reviews. JBI Evid Synth 2020;18:2108–18. 10.11124/JBISRIR-D-19-00169 32813460

[25] Thomas J , Harden A . Methods for the thematic synthesis of qualitative research in systematic reviews. BMC Med Res Methodol 2008;8:45. 10.1186/1471-2288-8-45 18616818 PMC2478656

[26] Barnett-Page E , Thomas J . Methods for the synthesis of qualitative research: a critical review. BMC Med Res Methodol 2009;9:59. 10.1186/1471-2288-9-59 19671152 PMC3224695

[27] Arvieux C , Laval G , Mestrallet JP , et al . [Treatment of malignant intestinal obstruction. A prospective study over 80 cases]. Ann Chir 2005;130:470–6. 10.1016/j.anchir.2005.05.011 16084483

[28] Cannizzaro R , Bortoluzzi F , Valentini M , et al . Percutaneous endoscopic gastrostomy as a decompressive technique in bowel obstruction due to abdominal carcinomatosis. Endoscopy 1995;27:317–20. 10.1055/s-2007-1005700 7555938

[29] Aría Guerra E , Cortés-Salgado A , Mateo-Lobo R , et al . Role of parenteral nutrition in oncologic patients with intestinal occlusion and peritoneal carcinomatosis. Nutr Hosp 2015;32:1222–7. 10.3305/nh.2015.32.3.9184 26319842

[30] Bozzetti F , Cozzaglio L , Biganzoli E , et al . Quality of life and length of survival in advanced cancer patients on home parenteral nutrition. Clin Nutr 2002;21:281–8. 10.1054/clnu.2002.0560 12135587

[31] Chermesh I , Mashiach T , Amit A , et al . Home parenteral nutrition (HTPN) for incurable patients with cancer with gastrointestinal obstruction: do the benefits outweigh the risks? Med Oncol 2011;28:83–8. 10.1007/s12032-010-9426-2 20107935

[32] Cotogni P , De Carli L , Passera R , et al . Longitudinal study of quality of life in advanced cancer patients on home parenteral nutrition. Cancer Med 2017;6:1799–806. 10.1002/cam4.1111 28557362 PMC5504329

[33] Abu-Rustum NR , Barakat RR , Venkatraman E , et al . Chemotherapy and total parenteral nutrition for advanced ovarian cancer with bowel obstruction. Gynecol Oncol 1997;64:493–5. 10.1006/gyno.1996.4605 9062158

[34] August DA , Thorn D , Fisher RL , et al . Home parenteral nutrition for patients with inoperable malignant bowel obstruction. JPEN J Parenter Enteral Nutr 1991;15:323–7. 10.1177/0148607191015003323 1907683

[35] Bond A , Teubner A , Taylor M , et al . A novel discharge pathway for patients with advanced cancer requiring home parenteral nutrition. J Hum Nutr Diet 2019;32:492–500. 10.1111/jhn.12650 31006921

[36] Brard L , Weitzen S , Strubel-Lagan SL , et al . The effect of total parenteral nutrition on the survival of terminally ill ovarian cancer patients. Gynecol Oncol 2006;103:176–80. 10.1016/j.ygyno.2006.02.013 16564074

[37] Chouhan J , Gupta R , Ensor J , et al . Retrospective analysis of systemic chemotherapy and total parenteral nutrition for the treatment of malignant small bowel obstruction. Cancer Med 2016;5:239–47. 10.1002/cam4.587 26714799 PMC4735773

[38] Duerksen DR , Ting E , Thomson P , et al . Is there a role for TPN in terminally ill patients with bowel obstruction? Nutrition 2004;20:760–3. 10.1016/j.nut.2004.05.010 15325683

[39] Dzierżanowski T , Sobocki J . Survival of patients with multi-level malignant bowel obstruction on total parenteral nutrition at home. Nutrients 2021;13. doi:10.3390/nu13030889. [Epub ahead of print: 10 Mar 2021]. PMC8000265 33801869

[40] Fan B-G . Parenteral nutrition prolongs the survival of patients associated with malignant gastrointestinal obstruction. JPEN J Parenter Enteral Nutr 2007;31:508–10. 10.1177/0148607107031006508 17947608

[41] Ethical dilemmas: extract from BMA’s draft handbook. BMJ 1979;1:1098–100. 10.1136/bmj.1.6170.1098 11663912 PMC1599475

[42] Keane N , Fragkos KC , Patel PS , et al . Performance status, prognostic scoring, and parenteral nutrition requirements predict survival in patients with advanced cancer receiving home parenteral nutrition. Nutr Cancer 2018;70:73–82. 10.1080/01635581.2018.1380206 29111787

[43] King LA , Carson LF , Konstantinides N , et al . Outcome assessment of home parenteral nutrition in patients with gynecologic malignancies: what have we learned in a decade of experience? Gynecol Oncol 1993;51:377–82. 10.1006/gyno.1993.1307 8112649

[44] Mercadante S . Parenteral nutrition at home in advanced cancer patients. J Pain Symptom Manage 1995;10:476–80. 10.1016/0885-3924(95)00053-2 7561231

[45] Patel PS , Fragkos KC , Keane N , et al . Clinical and nutritional care pathways of patients with malignant bowel obstruction: a retrospective analysis in a tertiary UK center. Nutr Cancer 2021;73:572–87. 10.1080/01635581.2020.1767165 32434435

[46] Ruggeri E , Giannantonio M , Agostini F , et al . Home artificial nutrition in palliative care cancer patients: impact on survival and performance status. Clin Nutr 2020;39:3346–53. 10.1016/j.clnu.2020.02.021 32143890

[47] Santarpia L , Alfonsi L , Pasanisi F , et al . Predictive factors of survival in patients with peritoneal carcinomatosis on home parenteral nutrition. Nutrition 2006;22:355–60. 10.1016/j.nut.2005.06.011 16413750

[48] Soo I , Gramlich L . Use of parenteral nutrition in patients with advanced cancer. Appl Physiol Nutr Metab 2008;33:102–6. 10.1139/H07-152 18347659

[49] Adelson MD , Kasowitz MH . Percutaneous endoscopic drainage gastrostomy in the treatment of gastrointestinal obstruction from intraperitoneal malignancy. Obstet Gynecol 1993;81:467–71. 7679789

[50] Brooksbank MA , Game PA , Ashby MA . Palliative venting gastrostomy in malignant intestinal obstruction. Palliat Med 2002;16:520–6. 10.1191/0269216302pm590oa 12465700

[51] Campagnutta E , Cannizzaro R , De Cicco M . Percutaneous endoscopic gastrostomy (PEG) in upper gastrointestinal tract obstructions in patients with gynecological cancer [Italian]. Minerva Ginecologica 1998;50:305–11. 9808954

[52] Cunningham MJ , Bromberg C , Kredentser DC , et al . Percutaneous gastrostomy for decompression in patients with advanced gynecologic malignancies. Gynecol Oncol 1995;59:273–6. 10.1006/gyno.1995.0021 7590486

[53] Diver E , O'Connor O , Garrett L , et al . Modest benefit of total parenteral nutrition and chemotherapy after venting gastrostomy tube placement. Gynecol Oncol 2013;129:332–5. 10.1016/j.ygyno.2013.02.002 23402902

[54] Dittrich A , Schubert B , Kramer M , et al . Benefits and risks of a percutaneous endoscopic gastrostomy (PEG) for decompression in patients with malignant gastrointestinal obstruction. Support Care Cancer 2017;25:2849–56. 10.1007/s00520-017-3700-1 28434096

[55] Gauvin G , Do-Nguyen CC , Lou J , et al . Gastrostomy tube for nutrition and malignant bowel obstruction in patients with cancer. J Natl Compr Canc Netw 2021;19:48–56. 10.6004/jnccn.2020.7604 33406493

[56] Goldberg JI , Goldman DA , McCaskey S , et al . Illness understanding, prognostic awareness, and end-of-life care in patients with Gi cancer and malignant bowel obstruction with drainage percutaneous endoscopic gastrostomy. JCO Oncol Pract 2021;17:e186–93. 10.1200/OP.20.00035 32758086 PMC8189623

[57] Herman LL , Hoskins WJ , Shike M . Percutaneous endoscopic gastrostomy for decompression of the stomach and small bowel. Gastrointest Endosc 1992;38:314–8. 10.1016/s0016-5107(92)70423-4 1607082

[58] Issaka RB , Shapiro DM , Parikh ND , et al . Palliative venting percutaneous endoscopic gastrostomy tube is safe and effective in patients with malignant obstruction. Surg Endosc 2014;28:1668–73. 10.1007/s00464-013-3368-7 24366189

[59] Jolicoeur L , Faught W . Managing bowel obstruction in ovarian cancer using a percutaneous endoscopic gastrostomy (PEG) tube. Can Oncol Nurs J 2003;13:212–9. 10.5737/1181912x134212215 14692364

[60] Kawata N , Kakushima N , Tanaka M , et al . Percutaneous endoscopic gastrostomy for decompression of malignant bowel obstruction. Dig Endosc 2014;26:208–13. 10.1111/den.12139 23772988

[61] Merchant SJ , Brogly SB , Booth CM , et al . Management of cancer-associated intestinal obstruction in the final year of life. J Palliat Care 2020;35:84-92. 10.1177/0825859719861935 31307272

[62] Pothuri B , Montemarano M , Gerardi M , et al . Percutaneous endoscopic gastrostomy tube placement in patients with malignant bowel obstruction due to ovarian carcinoma. Gynecol Oncol 2005;96:330–4. 10.1016/j.ygyno.2004.09.058 15661217

[63] Rath KS , Loseth D , Muscarella P , et al . Outcomes following percutaneous upper gastrointestinal decompressive tube placement for malignant bowel obstruction in ovarian cancer. Gynecol Oncol 2013;129:103–6. 10.1016/j.ygyno.2013.01.021 23369942 PMC4098040

[64] Scheidbach H , Horbach T , Groitl H , et al . Percutaneous endoscopic gastrostomy/jejunostomy (PEG/PEJ) for decompression in the upper gastrointestinal tract. initial experience with palliative treatment of gastrointestinal obstruction in terminally ill patients with advanced carcinomas. Surg Endosc 1999;13:1103–5. 10.1007/s004649901182 10556447

[65] Teriaky A , Gregor J , Chande N . Percutaneous endoscopic gastrostomy tube placement for end-stage palliation of malignant gastrointestinal obstructions. Saudi J Gastroenterol 2012;18:95–8. 10.4103/1319-3767.93808 22421713 PMC3326983

[66] Vashi PG , Braun DP , Popiel B , et al . Safety and efficacy of percutaneous endoscopic gastrostomy tube placement in patients with malignant peritoneal carcinomatosis induced bowel obstruction. Journal of Clinical Oncology 2012;30:e14012. 10.1200/jco.2012.30.15_suppl.e14012

[67] Zucchi E , Fornasarig M , Martella L , et al . Decompressive percutaneous endoscopic gastrostomy in advanced cancer patients with small-bowel obstruction is feasible and effective: a large prospective study. Support Care Cancer 2016;24:2877–82. 10.1007/s00520-016-3102-9 26838026

[68] Sowerbutts AM , Lal S , Sremanakova J , et al . Palliative home parenteral nutrition in patients with ovarian cancer and malignant bowel obstruction: experiences of women and family caregivers. BMC Palliat Care 2019;18:120. 10.1186/s12904-019-0507-5 31884962 PMC6936090

[69] Aramaki T , Arai Y , Takeuchi Y , et al . A randomized, controlled trial of the efficacy of percutaneous transesophageal gastro-tubing (PTEG) as palliative care for patients with malignant bowel obstruction: the JIVROSG0805 trial. Support Care Cancer 2020;28:07:07. 10.1007/s00520-019-05066-8 31494734

[70] Oh SY , Jun HJ , Park SJ , et al . A randomized phase II study to assess the effectiveness of fluid therapy or intensive nutritional support on survival in patients with advanced cancer who cannot be nourished via enteral route. J Palliat Med 2014;17:1266–70. 10.1089/jpm.2014.0082 24984081

[71] Higgins JPT TJ , Chandler J , Cumpston M . Cochrane Handbook for systematic reviews of interventions: cochrane, 2021.

[72] Sowerbutts AM , Lal S , Sremanakova J , et al . Discharging women with advanced ovarian cancer on home parenteral nutrition: making and implementing the decision. Nutrients 2020;12:7. 10.3390/nu12010166 PMC7019843 31936057

[73] Sowerbutts AM , Lal S , Sremanakova J , et al . Dealing with loss: food and eating in women with ovarian cancer on parenteral nutrition. J Hum Nutr Diet 2020;33:06:06. 10.1111/jhn.12738 32026525

[74] Singh Curry R , Evans E , Raftery A-M , et al . Percutaneous venting gastrostomy/gastrojejunostomy for malignant bowel obstruction: a qualitative study. BMJ Support Palliat Care 2019;9:381–8. 10.1136/bmjspcare-2019-001866 31597626

[75] Ziebland S , Chapple A , Evans J . Barriers to shared decisions in the most serious of cancers: a qualitative study of patients with pancreatic cancer treated in the UK. Health Expect 2015;18:3302–12. 10.1111/hex.12319 25496598 PMC5810685

[76] Charles C , Whelan T , Gafni A , et al . Doing nothing is no choice: lay constructions of treatment decision-making among women with early-stage breast cancer. Sociol Health Illn 1998;20:71–95. 10.1111/1467-9566.00081

[77] Patell R , Einstein D , Miller E , et al . Patient perceptions of treatment benefit and toxicity in advanced cancer: a prospective cross-sectional study. JCO Oncol Pract 2021;17:e119–29. 10.1200/OP.20.00517 33444075

[78] Weeks JC , Catalano PJ , Cronin A , et al . Patients' expectations about effects of chemotherapy for advanced cancer. N Engl J Med 2012;367:1616–25. 10.1056/NEJMoa1204410 23094723 PMC3613151

[79] Temel JS , Greer JA , Admane S , et al . Longitudinal perceptions of prognosis and goals of therapy in patients with metastatic non-small-cell lung cancer: results of a randomized study of early palliative care. J Clin Oncol 2011;29:2319–26. 10.1200/JCO.2010.32.4459 21555700

[80] Reyna VF , Nelson WL , Han PK , et al . Decision making and cancer. Am Psychol 2015;70:105–18. 10.1037/a0036834 25730718 PMC4347999

[81] Nierop-van Baalen C , Grypdonck M , van Hecke A , et al . Health professionals' dealing with hope in palliative patients with cancer, an explorative qualitative research. Eur J Cancer Care 2019;28:e12889. 10.1111/ecc.12889 30019789

[82] Bozzetti F , Arends J , Lundholm K , et al . ESPEN guidelines on parenteral nutrition: non-surgical oncology. Clin Nutr 2009;28:445–54. 10.1016/j.clnu.2009.04.011 19477052

[83] Arends J , Zuercher G , Dossett A . Non-surgical oncology - Guidelines on Parenteral Nutrition, Chapter 19. Nichtchirurgische Onkologie - Leitlinie Parenterale Ernährung, Kapitel 2009;19. 10.3205/000068 PMC2795366 20049066

[84] BIFA . The British intestinal failure alliance (BIFA) position statement palliative parenteral nutrition (HPN) for patients with malignancy, 2020. Available: https://www.bapen.org.uk/pdfs/bifa/position-statements/position-statement-on-palliative-hpn-for-patients-with-malignancy-dec-2020.pdf

[85] London AJ . Equipoise in research: integrating ethics and science in human research. JAMA 2017;317:525–6. 10.1001/jama.2017.0016 28170466

[86] Higginson IJ , Sen-Gupta GJ . Place of care in advanced cancer: a qualitative systematic literature review of patient preferences. J Palliat Med 2000;3:287–300. 10.1089/jpm.2000.3.287 15859670

[87] Anthony T , Baron T , Mercadante S , et al . Report of the clinical protocol Committee: development of randomized trials for malignant bowel obstruction. J Pain Symptom Manage 2007;34:S49–59. 10.1016/j.jpainsymman.2007.04.011 17544243

