# Supplementary material for: Inoperable malignant bowel obstruction: palliative interventions outcomes – mixed-methods systematic review
Source: BMJ Support Palliat Care. 2022 Jul 19;13(e3):e515–27. doi: 10.1136/bmjspcare-2021-003492 (PMC10850628; doi:10.1136/bmjspcare-2021-003492)
Supplement: Supplementary data [file bmjspcare-2021-003492supp001.pdf]

OVID FOR MEDLINE SEARCH STRATEGY

Parenteral nutrition

1 intestinal obstruction/ or duodenal obstruction/ or intestine obstruction/

2 ((bowel* or intestin* or gastrointestin* or gastro intestin* or colon* or colorect* or retrosigmoid*) adj3 (obstruct* or occlu* or block*)).ti,ab,kw.

3 1 or 2

4 exp Genital Neoplasm, Female/ or exp Ovarian Neoplasm/

5 ((ovar* or uterine or vaginal or vulva* or cervi* or gynae* or gyne*) adj3 (neoplasm* or tumor* or tumour* or cancer* or malignan* or carcinoma* or adenocarcinoma* or carcinosarcoma* or sarcoma*)).ti,ab,kw.

6 exp Intestinal Neoplasm/ or digestive system neoplasm/ or gastrointestinal neoplasm/ or peritoneal neoplasm/

7 ((neoplasm* or tumor* or tumour* or cancer* or malignan* or carcinoma* or adenocarcinoma* or carcinosarcoma* or sarcoma*) adj3 (rectal* or colorectal* or anal* or colo* or intestin* or abdominal or digest*)).ti,ab,kw.

8 4 or 5 or 6 or 7

9 Neoplasm/co or *Neoplasm/th

10 ((end stage* or incurable* or advanced*) adj3 cancer*).ti,ab,kw. or palliative care/ or terminal care/ or hospice care/

11 3 and 8 [obstruction and specific cancers]

12 malignan*.ti,ab,kw.

13 3 and 12 [obstruction and maligan*]

14 9 and 10 [cancer complications or focused cancer therapy]

15 11 or 13 or 14

16 exp Parenteral Nutrition/

17 Parenteral Nutrition, Home/

18 (total parenteral nutrition* or TPN* or parenteral nutrition* or PN*).mp.

19 ((parenteral* or artificial* or tub* or catheter* or intraven* or IV* or subcutan* or bypas*) adj3 (nutri* or hydration* or feed* or fed* or treatment* or manag* or method* or car* or support* or diet*)).mp.

20 parenteral nutrition.mp. or parenteral nutrition/ or nutrition supplement/

21 16 or 17 or 18 or 19 or 20 [parenteral nutrition]

22 15 and 21

Gastrostomy

1 intestinal obstruction/ or duodenal obstruction/ or intestine obstruction/

2 ((bowel* or intestin* or gastrointestin* or gastro intestin* or colon* or colorect* or retrosigmoid*) adj3 (obstruct* or occlu* or block*)).ti,ab,kw.

3 1 or 2

4 exp Genital Neoplasm, Female/ or exp Ovarian Neoplasm/

5 ((ovar* or uterine or vaginal or vulva* or cervi* or gynae* or gyne*) adj3 (neoplasm* or tumor* or tumour* or cancer* or malignan* or carcinoma* or adenocarcinoma* or carcinosarcoma* or sarcoma*)).ti,ab,kw.

6 exp Intestinal Neoplasm/ or digestive system neoplasm/ or gastrointestinal neoplasm/ or peritoneal neoplasm/

7 ((neoplasm* or tumor* or tumour* or cancer* or malignan* or carcinoma* or adenocarcinoma* or carcinosarcoma* or sarcoma*) adj3 (rectal* or colorectal* or anal* or colo* or intestin* or abdominal or digest*)).ti,ab,kw.

8 4 or 5 or 6 or 7

9 Neoplasm/co or *Neoplasm/th

10 ((end stage* or incurable* or advanced*) adj3 cancer*).ti,ab,kw. or palliative care/ or terminal care/ or hospice care/

11 3 and 8 [obstruction and specific cancers]

12 malignan*.ti,ab,kw.

13 3 and 12 [obstruction and maligan*]

14 9 and 10 [cancer complications or focused cancer therapy]

15 11 or 13 or 14

16 Gastrostomy/ or Gastroscopy/ or Jejunostomy/ or Gastrostomy*.mp. or Gastroscopy*.mp. or Jejunostomy*.mp. [mp=title, abstract, original title, name of substance word, subject heading word, floating sub-heading word, keyword heading word, organism supplementary concept word, protocol supplementary concept word, rare disease supplementary concept word, unique identifier, synonyms]

17 Decompression, Surgical/

18 Intubation, Gastrointestinal.mp. or Intubation, Gastrointestinal/

19 Intubation, Gastrointestinal/

20 gastrointestinal intubation tube/ or duodenum intubation/ or digestive tract intubation/ or stomach intubation/

21 ((gastrosom* or gastroscop* or jejunos* or percutan* or peg* or jej* or decomp* or intub*) adj3 (obstruct* or occlu* or block* or decomp* or intub*)).ti,ab,kw.

22 gastrostomy catheter/ or gastrostomy/ or percutaneous endoscopic gastrostomy tube/ or gastrostomy.mp. or percutaneous endoscopic gastrostomy/ or gastrostomy device/

23 Gastrostom$.mp.

24 16 or 17 or 18 or 19 or 20 or 21 or 22 or 23

25 15 and 24

OVID EMBASE SEARCH STRATEGY

Parenteral nutrition

1. intestinal obstruction/ or duodenal obstruction/ or intestine obstruction

2. ((bowel* or intestin* or gastrointestin* or gastro intestin* or colon* or colorect* or retrosigmoid*) adj3 (obstruct* or occlu* or block*)).ti,ab,kw.

3. 1 or 2

4. exp Genital Neoplasm, Female/ or exp Ovarian Neoplasm/

5. ((ovar* or uterine or vaginal or vulva* or cervi* or gynae* or gyne*) adj3 (neoplasm* or tumor* or tumour* or cancer* or malignan* or carcinoma* or adenocarcinoma* or carcinosarcoma* or sarcoma*)).ti,ab,kw.

6. exp Intestinal Neoplasm/ or digestive system neoplasm/ or gastrointestinal neoplasm/ or peritoneal neoplasm/

7. ((neoplasm* or tumor* or tumour* or cancer* or malignan* or carcinoma* or adenocarcinoma* or carcinosarcoma* or sarcoma*) adj3 (rectal* or colorectal* or anal* or colo* or intestin* or abdominal or digest*)).ti,ab,kw.

8. 4 or 5 or 6 or 7

9. Neoplasm/co or *Neoplasm/th

10. ((end stage* or incurable* or advanced*) adj3 cancer*).ti,ab,kw. or palliative care/ or terminal care/ or hospice care/

11. 3 and 8 [obstruction and specific cancers]

12. malignan*.ti,ab,kw.

13. 3 and 12 [obstruction and maligan*]

14. 9 and 10 [cancer complications or focused cancer therapy]

15. 11 or 13 or 14

16. exp Parenteral Nutrition/

17. Parenteral Nutrition, Home/

18. (total parenteral nutrition* or TPN* or parenteral nutrition* or PN*).mp.

19. ((parenteral* or artificial* or tub* or catheter* or intraven* or IV* or subcutan* or bypas*) adj3 (nutri* or hydration* or feed* or fed* or treatment* or manag* or method* or car* or support* or diet*)).mp.

20. 16 or 17 or 18 or 19 [parenteral nutrition]

21. 15 and 20

Gastrostomy

1 intestinal obstruction/ or duodenal obstruction/ or intestine obstruction/

2 ((bowel* or intestin* or gastrointestin* or gastro intestin* or colon* or colorect* or retrosigmoid*) adj3 (obstruct* or occlu* or block*)).ti,ab,kw.

3 1 or 2

4 exp Genital Neoplasm, Female/ or exp Ovarian Neoplasm/

5 ((ovar* or uterine or vaginal or vulva* or cervi* or gynae* or gyne*) adj3 (neoplasm* or tumor* or tumour* or cancer* or malignan* or carcinoma* or adenocarcinoma* or carcinosarcoma* or sarcoma*)).ti,ab,kw.

6 exp Intestinal Neoplasm/ or digestive system neoplasm/ or gastrointestinal neoplasm/ or peritoneal neoplasm/

7 ((neoplasm* or tumor* or tumour* or cancer* or malignan* or carcinoma* or adenocarcinoma* or carcinosarcoma* or sarcoma*) adj3 (rectal* or colorectal* or anal* or colo* or intestin* or abdominal or digest*)).ti,ab,kw.

8 4 or 5 or 6 or 7

9 Neoplasm/co or *Neoplasm/th

10 ((end stage* or incurable* or advanced*) adj3 cancer*).ti,ab,kw. or palliative care/ or terminal care/ or hospice care/

11 3 and 8 [obstruction and specific cancers]

12 malignan*.ti,ab,kw.

13 3 and 12 [obstruction and maligan*]

14 9 and 10 [cancer complications or focused cancer therapy]

15 11 or 13 or 14

16 Gastrostomy/ or Gastroscopy/ or Jejunostomy/ or Gastrostomy*.mp. or Gastroscopy*.mp. or Jejunostomy*.mp. [mp=title, abstract, original title, name of substance word, subject heading word, floating sub-heading word, keyword heading word, organism supplementary concept word, protocol supplementary concept word, rare disease supplementary concept word, unique identifier, synonyms]

17 Decompression, Surgical/

18 Intubation, Gastrointestinal.mp. or Intubation, Gastrointestinal/

19 Intubation, Gastrointestinal/

20 gastrointestinal intubation tube/ or duodenum intubation/ or digestive tract intubation/ or stomach intubation/0

21 ((gastrosom* or gastroscop* or jejunos* or percutan* or peg* or jej* or decomp* or intub*) adj3 (obstruct* or occlu* or block* or decomp* or intub*)).ti,ab,kw.

22 gastrostomy catheter/ or gastrostomy/ or percutaneous endoscopic gastrostomy tube/ or gastrostomy.mp. or percutaneous endoscopic gastrostomy/ or gastrostomy device/

23 Gastrostom$.mp.

24 16 or 17 or 18 or 19 or 20 or 21 or 22 or 23

25 15 and 24

CENTRAL SEARCH STRATEGY

Parenteral nutrition

#1 MeSH descriptor: [Intestinal Obstruction] explode all trees

#2 MeSH descriptor: [Duodenal Obstruction] explode all trees

#3 ((bowel* or intestin* or gastrointestin* or gastro intestin* or colon* or colorect* or retrosigmoid*) near/3 (obstruct* or occlu* or block*))

#4 #1 or #2 or #3

#5 MeSH descriptor: [Intestinal Neoplasms] explode all trees

#6 MeSH descriptor: [Gastrointestinal Neoplasms] explode all trees

#7 MeSH descriptor: [Peritoneal Neoplasms] explode all trees

#8 MeSH descriptor: [Digestive System Neoplasms] explode all trees

#9 MeSH descriptor: [Ovarian Neoplasms] explode all trees

#10 #5 or #6 or #7 or #8 or #9

#11 ((end stage* or incurable* or advanced*) near/3 (cancer or palliative care or terminal care or hospice care))

#12 ((neoplasm* or tumor* or tumour* or cancer* or malignan* or carcinoma* or adenocarcinoma* or carcinosarcoma* or sarcoma*) next/3 (rectal* or colorectal* or anal* or colo* or intestin* or abdominal or digest*))

#13 ((ovar* or uterine or vaginal or vulva* or cervi* or gynae* or gyne*) next/3 (neoplasm* or tumor* or tumour* or cancer* or malignan* or carcinoma* or adenocarcinoma* or carcinosarcoma* or sarcoma*))

#14 #11 or #12 or #13

#15 #10 or #14

#16 #4 and #15

#17 MeSH descriptor: [Parenteral Nutrition] explode all trees

#18 (total parenteral nutrition* or TPN* or parenteral nutrition* or PN*)

#19 ((parenteral* or artificial* or tub* or catheter* or intraven* or IV* or subcutan* or bypas*) near/3 (nutri* or hydration* or feed* or fed* or treatment* or manag* or method* or car* or support* or diet*))

#20 #17 or #18 or #19

#21 #16 and #20

Gastrostomy

#1 MeSH descriptor: [Intestinal Obstruction] explode all trees

#2 MeSH descriptor: [Duodenal Obstruction] explode all trees

#3 ((bowel* or intestin* or gastrointestin* or gastro intestin* or colon* or colorect* or retrosigmoid*) near/3 (obstruct* or occlu* or block*))

#4 #1 or #2 or #3

#5 MeSH descriptor: [Intestinal Neoplasms] explode all trees

#6 MeSH descriptor: [Gastrointestinal Neoplasms] explode all trees

#7 MeSH descriptor: [Peritoneal Neoplasms] explode all trees

#8 MeSH descriptor: [Digestive System Neoplasms] explode all trees

#9 MeSH descriptor: [Ovarian Neoplasms] explode all trees

#10 #5 or #6 or #7 or #8 or #9

#11 ((end stage* or incurable* or advanced*) near/3 (cancer or palliative care or terminal care or hospice care))

#12 ((neoplasm* or tumor* or tumour* or cancer* or malignan* or carcinoma* or adenocarcinoma* or carcinosarcoma* or sarcoma*) near/3 (rectal* or colorectal* or anal* or colo* or intestin* or abdominal or digest*))

#13 ((ovar* or uterine or vaginal or vulva* or cervi* or gynae* or gyne*) near/3 (neoplasm* or tumor* or tumour* or cancer* or malignan* or carcinoma* or adenocarcinoma* or carcinosarcoma* or sarcoma*))

#14 #11 or #12 or #13

#15 #10 or #14

#16 #4 and #15

#17 MeSH descriptor: [Gastrostomy] explode all trees

#18 MeSH descriptor: [Gastroscopy] explode all trees

#19 MeSH descriptor: [Intubation] explode all trees

#20 MeSH descriptor: [Intubation, Gastrointestinal] explode all trees

#21 MeSH descriptor: [Decompression, Surgical] explode all trees

#22 MeSH descriptor: [Enteral Nutrition] explode all trees

#23 Gastros*

#24 #17 or #18 or #19 or #20 or #21 #22 or #23

#25 #16 and #24

EBSCO FOR CINAHL COMPLETE

Parenteral nutrition

malignant bowel obstruction OR (advanced cancer or metastatic cancer or terminal cancer or palliative cancer or cancer) AND (parenteral nutrition or total parenteral nutrition or tpn)

Gastrostomy

malignant bowel obstruction OR (advanced cancer or metastatic cancer or terminal cancer or palliative cancer or cancer) AND gastrostomy

WEB OF SCIENCE

Parenteral nutrition

1 TOPIC: (Intestinal Obstruction* OR malignant bowel obstruction*)

2 TOPIC: (Parenteral nutrition)

3 #2 AND #1

Gastrostomy

1 TOPIC: (Intestinal Obstruction* OR malignant bowel obstruction*)

2 TOPIC: (Gastrostomy)

3 #2 AND #1

For BASE (Bielefeld Academic Search Engine), Caresearch (grey literature), ClinicalTrials.gov, EU Clinical Trials Register and the World Health Organization (WHO) International Clinical Trials Registry Platform (ICTRP) we combined terms for malignant bowel obstruction AND parenteral nutrition or gastrostomy.

Searches prior to deduplication.

**Database**	**Parenteral nutrition (2020)**	**Gastrostomy (2020)**	**Parenteral nutrition (2021)**	**Gastrostomy (2021)**
Medline	730	808	56	54
Embase	1394	1243	51	76
Central	148	32	5	1
CINHAL	1212	111	64	49
Web of science	458	181	28	11
Caresearch	269	15	0	0
BASE	515	69	38	0
Clinicaltrials.gov	224	10	0	0
EU Clinical Trials Register	21	16	0	0
WHO ICTRP	9	13	0	0
Citation Searches of Selected key studies of particular importance= 136				
