# Supplementary material for: Inoperable malignant bowel obstruction: palliative interventions outcomes – mixed-methods systematic review
Source: BMJ Support Palliat Care. 2022 Jul 19;13(e3):e515–27. doi: 10.1136/bmjspcare-2021-003492 (PMC10850628; doi:10.1136/bmjspcare-2021-003492)
Supplement: Supplementary data [file bmjspcare-2021-003492supp002.pdf]

BMJ Publishing Group Limited (BMJ) disclaims all liability and responsibility arising from any reliance placed on this supplemental material which has been supplied by the author(s)

CASP COHORT STUDY CHECKLIST

STUDY	1	2	3	4	5a	5b	6a	6b	7	8	9	10	Comments
**Abu-Rustum 1997**	?	●	●	●	×	×	●	●	?	×	●	×	unclear aim, risk of biases, risk of confounding, data presentation
**Aria Guerra 2015**	●	●	●	●	×	●	●	●	●	●	●	●	risk of biases, risk of confounding,
**August 1991**	●	?	×	×	×	×	●	●	?	?	?	?	risk of biases, risk of confounding, data presentation
**Bond 2019**	?	●	●	●	×	×	●	●	●	●	●	?	risk of biases, risk of confounding
**Bozzetti 2002**	●	×	●	●	×	●	●	●	●	●	●	●	risk of biases, risk of confounding,
**Brard 2006**	●	×	●	●	×	×	●	●	●	●	?	×	risk of confounding, risk of selection bias,
**Chermesh 2011**	?	●	?	?	×	×	●	●	●	×	●	●	unclear aim, risk of confounding, data presentation
**Chouhan 2016**	●	●	●	●	●	×	●	●	●	●	●	●	risk of confounding,
**Cotogni 2017**	●	●	●	●	●	●	●	●	●	●	●	?	
**Duerksen 2004**	●	●	×	●	×	×	●	●	●	×	●	?	risk of selection bias, risk of confounding, data presentation
**Dzierianowski 2021**	●	●	×	●	●	●	●	●	●	●	●	●	risk of selection bias,
**Fan 2007**	?	●	×	●	×	×	●	●	●	×	●	●	risk of selection bias, risk of confounding, data presentation
**Keane 2018**	●	●	?	●	●	●	●	●	●	●	●	●	risk of selection bias
**King 1993**	●	●	●	×	×	×	●	●	●	●	●	●	risk of reporting bias, risk of confounding,
**Mercadante 1995**	?	?	●	●	×	×	●	●	×	×	●	●	unclear aim, risk of biases, risk of confounding, data presentation
**Patel 2021**	●	●	?	?	●	●	●	●	●	●	●	●	risk of bias, risk of confounding,
**Ruggeri 2020**	●	●	●	●	×	×	●	●	●	●	●	●	risk of bias, risk of confounding,
**Santarpia 2006**	?	●	●	●	●	●	×	×	●	●	●	●	unclear aim, risk of bias
**Soo 2018**	●	●	●	●	×	×	●	?	●	×	●	●	
**Adelson 1993**	?	?	?	×	×	×	●	●	?	×	●	×	unclear aim, risk of biases, risk of confounding, data presentation
**Arvieux 2005**	●	●	?	?	×	×	●	●	?	?	?	×	risk of biases, risk of confounding, unclear data presentation
**Brooksbank 2002**	?	●	●	×	×	×	●	●	?	×	●	●	unclear aim, risk of biases, risk of confounding,
**Cannizzaro 1995**	●	●	●	●	×	×	?	?	●	?	●	●	risk of biases, risk of confounding,
**Champagnutta 1998**	●	●	?	●	×	×	?	?	●	●	●	●	risk of biases, risk of confounding,
**Cunningham 1995**	?	●	●	×	×	×	?	●	?	?	●	●	unclear aim, risk of biases, risk of confounding, unclear data presentation
**Diver 2013**	?	●	●	●	×	×	●	●	●	●	●	●	risk of biases, risk of confounding,

**Dittrich 2017**	●	×	●	?	●	●	●	●	●	●	●	●	risk of biases,
**Gauvin 2021**	●	?	●	●	×	×	●	●	●	●	●	●	risk of confounding,
**Goldberg 2021**	●	●	●	?	×	×	●	●	●	●	●	●	risk of measurement bias, risk of confounding,
**Herman 1992**	?	●	×	×	×	×	●	●	?	?	●	●	risk of biases, risk of confounding, data presentation
**Issaka 2014**	●	?	●	×	×	×	●	●	●	●	●	●	risk of confounding,
**Jolicoeur 2003**	●	?	●	●	×	×	●	●	?	?	●	●	risk of biases, risk of confounding, unclear data presentation
**Kawata 2014**	●	?	●	×	×	×	?	●	●	●	●	●	risk of reporting biases, risk of confounding,
**Lilley 2018**	●	?	●	●	●	●	●	●	●	●	●	?	risk of selection bias
**Merchant 2020**	●	●	●	●	×	●	●	●	●	●	●	●	risk of confounding,
**Pothuri 2005**	●	●	●	●	×	×	●	●	●	●	●	●	risk of biases, risk of confounding
**Rath 2013**	●	●	●	●	×	×	●	●	●	●	●	●	risk of biases, risk of confounding
**Scheidbach 1999**	?	●	●	×	×	×	●	●	●	●	●	●	risk of biases, risk of confounding,
**Teriaky 2012**	●	●	●	●	×	×	?	×	?	●	×	●	risk of biases, risk of confounding, unclear data presentation
**Vashi 2012**	?	●	●	×	×	×	?	?	●	?	●	●	unclear aim, risk of biases, risk of confounding, unclear data presentation
**Zucchi 2016**	●	●	●	●	×	×	●	●	●	●	●	●	risk of confounding,

CASP QUALITATIVE STUDIES CHECKLIST

STUDY	1	2	3	4	5	6	7	8	9	10	Comments
**Sowerbutts 2019**	•	•	•	•	•	?	•	•	•	•	unclear reflexivity in research design,
**Sowerbutts 2020a**	•	•	×	•	•	×	•	•	•	•	unclear reflexivity in research design, unclear research design justification
**Sowerbutts 2020b**	•	•	•	•	•	?	•	•	•	•	unclear reflexivity in research design,
**Singh curry 2019**	•	•	•	•	•	×	•	•	•	•	unclear reflexivity in research design,

ROB 2: A REVISED COCHRANE RISK-OF-BIAS TOOL FOR RANDOMIZED TRIALS

**Oh 2014**		
**Bias**	**Authors' judgement**	**Support of judgement**
Random sequence generation (selection bias)	Low risk	Permuted-block randomization
Allocation concealment (selection bias)	Low risk	Allocated group was announced to investigators at the time of assignment of each patient by telephone call
Blinding of participants and personnel (performance bias) All outcomes	Unclear risk	Insufficient information to permit judgement
Blinding of outcome assessment (detection bias) All outcomes	Unclear risk	Insufficient information to permit judgement
Incomplete outcome data (attrition bias) All outcomes	High risk	The study was closed early because of poor patient accrual, and it was expected that the target population could not be achieved within the study time frame.
Selective reporting (reporting bias)	Low risk	No evidence of selective reporting.
Other bias	Unclear risk	Possible inclusion bias

**Aramaki 2019**		
**Bias**	**Authors' judgement**	**Support of judgement**
Random sequence generation (selection bias)	Unclear risk	Insufficient information to permit judgement
Allocation concealment (selection bias)	Unclear risk	Insufficient information to permit judgement
Blinding of participants and personnel (performance bias) All outcomes	Unclear risk	Open -but assessor(s) are blinded
Blinding of outcome assessment (detection bias) All outcomes	Unclear risk	Insufficient information to permit judgement
Incomplete outcome data (attrition bias) All outcomes	Low risk	No missing outcome data
Selective reporting (reporting bias)	Unclear risk	Insufficient information to permit judgement
Other bias	Low risk	The study appears to be free of other sources of bias
