# Supplementary material for: Inoperable malignant bowel obstruction: palliative interventions outcomes – mixed-methods systematic review
Source: BMJ Support Palliat Care. 2022 Jul 19;13(e3):e515–27. doi: 10.1136/bmjspcare-2021-003492 (PMC10850628; doi:10.1136/bmjspcare-2021-003492)
Supplement: Supplementary data [file bmjspcare-2021-003492supp003.pdf]

Characteristics of Included Studies

Author, Year, Study Design and Location	Study Aim	Sample Size, Age, Sex and % with MBO	Cancer Diagnosis and Treatment	Definition of survival	Survival	HRQoL
Parenteral Nutrition						
Abu-Rustum 1997 Retrospective cohort study USA	To determine the efficacy of intravenous chemotherapy alone or with PN in restoring bowel function	21<br><br>Mean 54.5 years (range 32 to 75)<br><br>*F* n= 21<br><br>100%	*Diagnosis*<br>Advanced ovarian cancer<br>*Treatment*<br>Chemotherapy with a median of three regimens prior to developing intestinal obstruction (range two to six regimens).	From venting gastrostomy insertion	Median for those who received salvage chemotherapy was 89 days, longer than for patients who received salvage chemotherapy alone (71 days) (P =0.031).	NR
AriaGuerra 2015 Prospective cohort study Spain	Aimed to analyse the effects of parenteral nutrition in oncologic patients with intestinal occlusion and peritoneal carcinomatosis regarding prognosis	55<br><br>60±13 years<br>Sex- NR<br><br>100%	*Diagnosis*<br>• Gastrointestinal tumours n= 38<br>• Gynaecological tumours n= 10<br>• Other n= 7<br><br>*Treatment*- NR	From starting PN to death	Median 40 days (range 2-702)	NR
August 1991 Retrospective cohort study USA	To review the Yale-New Haven Hospital experience with HPN in MBO	18<br>median 58 years (range 33 to 79)<br>*F* n= 13	*Diagnosis*<br><br>• Ovarian n=9<br>• Colon n=4<br>• Endometrium n= 1	From discharge to death	Median 53 days (range 5-208 days)	For 11 patients all agreed the HPN was beneficial or highly beneficial. In three patients

	patients to determine the efficacy, safety, and indications for HPN in this patient population.	*M* n= 4<br><br>100%	• Appendix n= 2<br>• Stomach n= 1<br><br>*Treatment*- NR			the therapy was not beneficial.
Bond 2019 Retrospective cohort study UK	To assess the impact of a novel remote discharge pathway for palliative HPN patients	82<br>Mean 57 (range 24–80)<br>*F* n= 66<br>*M* n= 16<br><br>100%	*Diagnosis*<br>• Ovarian n= 41<br>• Peritoneal n= 7<br>• Colorectal n= 7<br>• Gastric n= 5<br>• Lymphoma n= 2<br>• Neuroendocrine tumour n= 4<br>• Pseudomyxoma n= 4<br>• Breast n= 3<br>• Endometrial n= 3<br>• Bladder n= 3<br>• Unknown n= 2<br>• Sarcoma n= 1<br>*Treatments-NR*	From discharge to death	Mean 134.8 days(1–1715 days)	NR
Bozzetti 2002 Prospective cohort study Italy	To investigate changes in the quality of life in cancer patients during HPN and to determine whether it is possible to predict length of survival before administering HPN	69<br><br>Mean 54 years (range 29–82)<br><br>*F* n= 28<br>*M* n= 41<br><br>84%	*Diagnosis*<br>• Colorectal n= 21<br>• Stomach n= 16<br>• Uterus/ovary n= 13<br>• Breast n= 2<br>• Other n= 17<br><br>*Treatment*- NR	From starting PN to death	Median 4 months (range 1–14)	Rotterdam symptom checklist (RSCL)- regards to physical, psychological and activity assessment, roughly half patients deteriorated and 40% improved

						with a small percentage of patients who showed no change. In contrast, only one-fourth of patients showed a worsening of the well-being assessment. The median changes were not significantly different from 0 for all the assessments.
Brard<br>2006<br>Retrospective cohort study<br>USA	The goal of this retrospective cohort study was to investigate the role of TPN in terminally ill epithelial ovarian cancer patients with terminal intestinal obstruction (TIO) and its effects on survival after TIO diagnosis and any potential complications of this therapy	55<br><br>Mean 56.4 years (SD 11.7)<br><br>*F* n= 55<br><br>100%	*Diagnosis*<br><br>Patients with stage IIIC/IV epithelial ovarian cancer<br><br>*Treatment*<br><br>All patients were previously treated with paclitaxel/platinum following cytoreductive surgery.	From time of MBO diagnosis	Patients survived a median of 72 days (range 16–485) if they received TPN compared to 41 days (range 4–133) if no TPN was administered. The mortality rate ratio for TPN users compared to non-users was 0.59 (95% CI: 0.35–1.00).	NR

Chermesh 2011 Prospective cohort study Isreal	To define the role of PN in patients with MBO	28<br><br>Mean 59.9 ± 12.7 years<br><br>F n= 13<br>M n=15<br><br>82%	Diagnosis<br><br>• Ovary n= 9<br>• Stomach n= 8<br>• Colon n= 4<br>• Pancreas n= 3<br>• Breast n= 2<br>• Squamous cell carcinoma of the larynx presumed n= 1<br>• Carcinoid presumed n = 1	Not defined	Median 140 days (range 20–783) with no difference with regard to age, gender, primary diagnosis, BMI, percentage of weight loss, albumin level and occurrence of TPN-related complications.<br><br>Patients with KPS > 50 had significantly longer survival than patients with KPS < 50 62 days (range 20–141) vs 211 days (range 50–783).	NR
Chouhan 2016 Retrospective cohort study USA	To examine a large, single-institution dataset to describe outcomes associated with concurrent TPN and systemic chemotherapy for persistent MSBO after conservative management.	82<br><br>Median 55 years (range 17–85)<br><br>*F* n= 51<br>*M* n= 31<br><br>100%	*Diagnosis*<br><br>• Gastrointestinal n= 49<br>• Gynecological n= 18<br><br>• Other n= 15<br><br>*Treatment*<br><br>Chemotherapy<br>1st line 38 (46.3%)<br>2nd line 15 (18.3%)<br>≥3rd line 29 (35.3%)<br><br>New chemotherapy start during TPN- 58 (70.7%)	From the initial date where TPN supplementation and chemotherapeutic intervention coincided	Median survival 3.1 months (range, 0.03–69.4 months), with a 1-year overall rate of 12.2%. The median duration of TPN was 45 days (range, 9–639)	NR
Cotogni 2017	To analyse the quality of life in advanced cancer	111	*Diagnosis*<br><br>• Stomach n= 38	From discharge	Median 4.7 months, (range 1–42)	Patients with advanced malignancy

Prospective cohort study Italy	patients on HPN, and to investigate whether the combination with oncologic treatments correlates with changes in quality of life.	Median 62 years (range 32 to 79)<br><br>*F* n= 54<br>*M* n= 57<br><br>90%	• Colon/rectum n= 21 Pancreas/biliary system n= 20<br>• Oesophagus n= 10<br>• Lung n= 10<br>• Ovary n= 2<br>• Others n= 10<br><br>*Treatment*<br><br>Chemotherapy n= 61 Radiation therapy n= 2<br>Both treatments n= 9	with HPN to death		requiring a nutritional supplementation through HPN maintained their QoL or even showed an improvement in some scores according to the EORTC QLQ-C30. The items which significantly improved were the domains of global QoL, physical, role, and emotional functioning, as well as appetite loss and fatigue.
Deurksen 2004 Retrospective cohort study Canada	The objective of this study was to determine whether a subgroup of patients with intestinal obstruction would benefit from support with TPN, identify factors that might predict patients who would benefit from home	9<br><br>Mean- 45 (range 35 to 57)<br><br>*F* n= 3<br>*M* n= 6<br><br>100%	*Diagnosis*<br><br>• Gastric n= 4<br>• Colon n= 4<br>• Cholangiocarcinoma n= 1<br><br>*Treatment*- NR	From starting PN to death	Median 84 (range 26 to 433 d)	NR

	TPN and identify issues relevant for prospective study.					
Dzierianowski 2021<br>Retrospective cohort study<br>Poland	To verify the overall survival and impact of the overall performance status, clinical symptoms, and laboratory test results at HPN initiation on patients' survival probability with MBO	114<br><br>Mean (95% CI)- 54.7 (52.5–56.9)<br><br>*F* n= 81<br>*M* n= 33<br><br>100%	*Diagnosis*<br><br>• Colorectal n= 19<br>• Stomach n= 40<br>• Other gastroenterological n= 7<br>• Gynecological n= 33<br>• Ovarian n= 25<br>• Other gynecological n= 8<br>• Other n= 15<br><br>*Treatment-* NR	From starting PN to death	Median (Q25–Q75) 89 (52–186) (range, 16–1393)<br><br>Survival based on ECOG<br>• 0 Median 680 (range 543–1393)<br>• 1 Median 174 (range 65–748)<br>• 2 Median 61.5 (range 25–399)<br>• 3 Median 26 (range 16–64)	NR
Fan<br>2007<br>Retrospective cohort study<br>China	The purpose of the study was to explore life expectancy in the patient with advanced cancer who received PN after cessation of energy intake due to malignant GI tract obstruction.	115<br><br>Mean- 51 (range, 31–74)<br>*F* n= 62<br>*M* n= 53<br><br>100%	*Diagnosis*<br><br>• Gastric carcinoma n= 24<br>• Colorectal carcinoma n= 23<br>• Oesophageal carcinoma n= 20<br>• Jejunal carcinoma n= 14<br>• Breast carcinoma n= 10<br>• Sarcoma n= 9<br>• Cholangiocarcinoma n= 9<br>• Pancreatic carcinoma n= 3<br>• Lymphoma n= 3	From the initiation of PN to death	Mean 6.5 months<br><br>Eleven patients survived ≥1 year and 2 patients have been alive for almost 4 years later after cessation of energy intake.	NR

			Treatment- NR			
Keane<br>2018<br>Retrospective cohort study<br>UK	To examine the prognostic significance of performance status, type and site of tumour, previous or concurrent chemo-radiotherapy, anthropometric characteristics, nutritional and inflammatory status, demographic characteristics, serum biochemistry, and prognostic indices based on a large cohort of patients with advanced cancer receiving HPN at University College London Hospitals	107<br><br>Mean age 57 ± 12 years<br>*F* n= 68<br>*M* n= 39<br><br>74.3%	*Diagnosis*<br><br>• Gynaecological n= 37<br>• Upper Gastrointestinal n= 21<br>• Lower Gastrointestinal n= 24<br>• Hepato-pancreatobiliary n= 10<br>• Haematological n= 5<br>• Other n= 10<br><br>Most patients had metastatic disease (81.3%)<br><br>*Treatments*<br><br>Most patients had undergone surgery for their malignancy (79%), or chemotherapy before and/or during PN administration (90.4%). The majority of patients were radiotherapy naive (71.2%).	Measured from discharge until death	Overall mean survival was 30.8 weeks (95% CI 21.4–39.6) and median survival was 14 weeks (IQR 5–34).	NR
King<br>1993<br>Retrospective cohort study<br>USA	1) Review our experience of in gynaecological cancer patients who received HPN.<br>2) determine if	61<br><br>Age- mean 55.0 years.<br>*F* n= 61	*Diagnosis*<br>• Ovarian n= 34<br>• Cervix n= 15<br>• Corpus n= 9<br>• Vulva n= 2<br>• Vagina n =1	Date of initiation of HPN to last follow-up or death	Mean 167.5 days, median 60 days (range 2-780 days)	Prior to HPN starting versus during HPN<br>KPS 48 47

	HPN improved patients nutrition parameters, survival and quality of life	72%	*Treatment*<br>Surgery n= 60<br>Chemotherapy n= 56<br>Radiotherapy n= 43<br>30 patients had been treated with all three modalties, and 27 had been treated with two modalities.<br><br>*Treatment received during HPN*<br>Surgery n= 14<br>Chemotherapy n= 31<br>Radiotherapy n= 7<br>*Doesn't state surgery for resolution of MBO.			Activity level<br>3.8<br>3.5<br>Pain 2.6<br>2.3<br>GI discomfort<br>2.8<br>2.4<br>N & V 3.2<br>2.7<br>Fatigue 3.4<br>3.0<br>Diarrhoea<br>2.0<br>1.8<br>Morale 2.7<br>2.5<br>Social interaction family/friends<br>2.8<br>2.5<br>Note, 1, usual or best; 5, worst or never.
Mercadante 1995<br>Retrospective cohort study<br>Italy	To describe clinical experience with HPN patients	13<br><br>Age- mean 56 years (32 to 71)<br>*F* n= 8<br>*M* n= 5<br><br>100%	*Diagnosis*<br>• Pharynx n= 1<br>• Colon n= 4<br>• Stomach n= 1<br>• Breast n 1<br>• Ileum n 2<br>• Ovary n 2<br>• Oesophagus n= 1<br>• Pancreas n= 1	From the initiation of PN to death	Mean 30.4 days (range 3-121 days)	NR

			Treatments- NR			
Oh<br>2014<br>Randomised control trial<br>South Korea	To investigate the effect of PN on prolonging survival at the end of life in patients with terminal cancer	16<br><br>Age (years)<br>60.4 ± 12.6<br>*F* n= 6<br>*M* n= 10<br><br>100%	Diagnosis<br><br>• Hepatobiliary and pancreas n= 2<br>• Colon n= 4<br>• Stomach n= 4<br>• Breast n= 1<br>• Neuroendocrine n= 2<br>• Lung n= 1<br>• Prostate n= 1<br>• Salivary gland n= 1<br><br>*Treatments*- NR	Survival was defined as the time from randomisation to death or to withdrawal from the study.	Median survival of the PN group was 13 days (95% CI, 3.1–22.9 days)<br>median survival of the control group was 8 days (95% CI, 5.7–10.3 days)	NR
Patel<br>2021<br>Retrospective cohort study<br>UK	To examine i) what characterizes the MBO population, ii) what medical and nutritional care do patients with MBO who are referred or not referred for nutrition receive and iii) if any of these care pathways affect survival.	72<br><br>mean (SD)<br>63.1 (13.1) years<br><br>*F* n= 57<br>*M* n= 15<br><br>100%	*Diagnosis*<br><br>• Gynaecology n = 36<br>• Lower GI n= 19<br>• Upper GI n= 3<br>• HPB n= 3<br>• Urology n= 2<br>• Haematology n= 1<br>• Breast n= 1<br>• Other n= 6<br><br>*Treatment*<br><br>Prior surgery for cancer n= 32<br>Prior radiotherapy n= 17<br>Prior chemotherapy n= 52	From admission with MBO to death or censorship	Median (range) 20 (5.9–65.1) weeks (4.7 (1.4–15.2) months. There was a survival advantage in those in the HPN group vs. those who may have required PN group (323 vs. 91 day, respectively P= 0.0021).	NR
Ruggeri<br>2020<br>Retrospective cohort study	To describe the selection criteria used for identifying the eligible patients	629<br><br>mean ± SD<br>64.2 ± 12.6	*Diagnosis*<br><br>• Gastrointestinal tract n= 319	Date of initiation of HPN to last	Survival (weeks) (n= 564) - Mean (SD) 16.1 (18.0), median (95% C.I.) (9.0-11.3)	NR

Italy	for home artificial nutrition (HAN), and to evaluate the impact of HAN on performance status and survival in cancer patients assisted at home by a palliative care program.	*F* n= 305<br>*M* n= 324<br><br>77.8%	• Head-neck n= 104<br>• Other organs n= 114<br>• Lung n= 20<br><br>*Treatments*- NR	follow-up or death	KPS at the entry was significantly associated with estimated survival time: a higher KPS at the start of HAN predicted a longer survival [odds ratio 1⁄4 0.9, p < .001,] HPN not separated out from HAN as a whole.	
Santarpia 2006 Retrospective cohort study Italy	To identify predictors of survival in patients with carcinomatosis on home parenteral nutrition	152<br><br>Mean 57.8 +/- 13.6 years. Median 59.5 years (Range: 22.0 - 88.0 years)<br>*F* n= 107<br>*M* n= 45<br><br>100%	*Diagnosis*<br><br>• Stomach n=48<br>• Ovarian n= 42<br>• Colorectal n = 30<br>• Endometrium n = 7<br>• Breast n = 6<br>• Iluem n = 5<br>• Gallbladder n = 4<br>• Pancreas n = 3<br>• Kidney n = 2<br>• Skin n = 1<br>• Prostate n = 1<br>• Abdominal sarcoma n = 1<br>• Unknown n = 2<br><br>*Treatments*- "These patients were considered terminal because they were unresponsive to any oncologic treatment"	Date of initiation of HPN to death	Median 45 days (range, 6–1269).	NR
Soo 2018	To describe the patient-related	38	*Diagnosis*	Not specified	Mean survival 5.4 months (range 0.25–33).	NR

Retrospective cohort study<br>Canada	variables in a cohort of advanced cancer patients (ACPs) enrolled in a HPN program	48.76 years (SD 13.8)<br>*F* n= 27<br>*M* n= 11<br><br>84.2%)	• Ovarian n=13<br>• Colonic n=6<br>• Gastric n=6<br>• Peritoneal n=3<br>• Unknown n=2<br>• Oesophageal n=2<br>• Carcinoid n=1<br>• Cervical n=<br>• Ampullary n=1<br>• GIST n=1<br>• Anaplastic large-cell lymphoma n=1<br>• Rectal n=1<br><br>*Treatment*<br><br>Chemotherapy n=14<br>Chemotherapy and radiotherapy n=1<br>None n = 23		Start of HPN KPS > 50 (median 70, IQR 68.75 - 81.86), had a 6-month median duration of life (IQR 2.75 - 9.5). Start of HPN KPS <50 (median 50, IQR 45 - 50), had a median survival of 3 months (IQR 1.75-3.5), p=0.02; two-tailed.	
Sowerbutts 2019<br>Mixed-methods study<br>UK	To investigate the experience of HPN for women with ovarian cancer and MBO and their family members acting as caregivers, in the context of the nutritional status and survival of a cohort of patients	38<br><br>Age, mean ± SD- Interviewed 67 (7.5), not interviewed 64 (10.1)<br>*F* n=38<br><br>100%	*Diagnosis*<br><br>All patients diagnosed with ovarian cancer.<br><br>*Treatment*- NR	From admission with MBO	Median for all 38 women was 70 days (range 8 to 506).<br>Median for 32 women who received PN was 81 days (range 10 to 506).<br>Median for the 17 patients who had HPN was 156 days (range 46–506).<br>Median for 6 women who did not receive PN was 20 days (range 8 to 109).	Qualitative synthesis.

	with ovarian cancer and MBO.					

Author, Year, Study Design and Location	Study Aim	Sample Size, Age, Sex and % with MBO	Cancer Diagnosis, Treatment	Definition of survival	Survival	Symptoms	HRQoL
Palliative Venting Gastrostomy							
Aramaki 2019 Randomised control trial Japan	To evaluate the superiority of PTEG over NGT as palliative care for bowel obstruction in patients with terminal malignancy from the perspective of patient QOL	40 (21 PEG, 19 NGT)<br><br>Mean 59.3, Median 32 (Range 34-76) F n= 14 M n= 25<br><br>100%	*Diagnosis*<br><br>21 PTEG group<br>• Colorectal n= 10<br>• Stomach n= 6<br>• Ovarian n= 2<br>• Bile duct n= 2<br>• Pancreatic n= 1<br>19 NGT group<br>• Stomach n =6<br>• Colorectal n= 3<br>• Pancreatic n= 3<br>• Ovarian n= 3<br>• Peritoneal mesothelioma n= 2<br>• Oesophageal n= 1<br>• Bile duct n= 1<br>• Unknown primary n= 1<br><br>*Treatment*- NR	From gastrostomy placement	50 days for the PEG group and 86 days for the NGT group	Included in HRQoL	Mean EQ-5D scores for the PTEG and NGT groups were 7.132 (4.543–9.702) and 3.663 (0.464–6.862), respectively. Mean SF-8 scores were 420.1 (282.6–557.6) and 199.4 (22.2–376.6), respectively.
Adelson 1993	Evaluate the effectiveness of a percutaneous	13	Diagnosis<br><br>• Ovarian n= 9	Length of tube placement	median 62 days (range 5-246) one	All gastrostomy tubes provided	NR

Retrospective cohort study USA	technique for placement of a drainage gastrostomy.	Median-61 (range 42-78)<br>*F* n= 13<br><br>100%	• Cervical n =2<br>• Papillary peritonela serous tumour n= 1<br>• Breast n= 1<br><br>*Treatment*<br><br>Prior laparotomies median two (range 1-4)		tube removed electively 131 days after resolution of SBO	GI driainage and relief of n&v and abdo pain due to distention.	
Arvieux 2005 Prospective cohort study France	To draw up a specific medicosurgical protocol for immediate response to the start of bowel obstruction in cancer patients with end-stage peritoneal carcinomatosis who cannot receive curative treatment.	10<br><br>Mean 57.9 years (median at 62.5, range 22–84)<br>F n= 8<br>M n= 2<br><br>100%	*Diagnosis*<br><br>• Ovarian n= 6<br>• Pancreas n=1<br>• Stomach n=1<br>• Bladder n=1<br>• Melanoma n=1<br><br>*all causing carcinamatosis.	From gastrostomy placement	median 13 days (range 6 to 125 days)	100% relief of symptoms	NR
Brooksbank 2002 Retrospective cohort study Australia	An update of our preliminary experience with palliative venting gastrostomy (PVG), which was first reported in 1991	51<br><br>61 years (range 25±86 years)<br>*F* n= 32<br>*M* = n 19	*Diagnosis*<br><br>• Colon and rectum n= 27<br>• Ovary n= 16<br>• Breast n= 2<br>• Pancreas n= 2<br>• Other n= 4<br><br>*Treatment*<br><br>All had previous surgery. All patients had been treated with various first-line anti-emetic agents,	From gastrostomy placement	median survival 17 days (range 1±190)	For 47/51 (92%) patients, the symptoms of nausea and vomiting were substantially or completely relieved	Many patients were also able to resume some degree of oral intake of soft food and drink. This was usually seen by both patients and families as a

			mainly metoclopramide, haloperidol and prochloperazine, alone or in combination. Dexamethasone was used in six patients. Octreotide was used in three patients.				positive outcome.
Cannizzaro 1995 Prospective cohort study Italy	To assess their experience in performing endoscopic gastrostomy in patients with obstructing disseminated abdominal cancers, also compared the efficacy of two catheters of different sizes, 15 and 20 Fr respectively, in obtaining symptomatic relief.	22<br><br>Mean 53.7 (range 29-73)<br>*F* n= 22<br><br>100%	*Diagnosis*<br><br>• Ovarian n= 14<br>• Endometrial n =5<br>• Colon n= 3<br><br>*Treatment*<br><br>Previous abdominal surgery reported for all participants.	From gastrostomy placement	Mean 74 days (Range 13-272)	100% of patient had reduced symptoms. 100% of patients tolerated soft and liquid foods after PEG placement.	NR
Champagnutta 1998 Retrospective cohort study Italy		64<br><br>56 (range 20- 76) years<br>*F* n= 56<br><br>100%	*Diagnosis*<br><br>• Colon n= 9<br>• Stomach n = 2<br>• Gallbladder n= 2<br>• Breast n= 1<br>• Ovarian n =41<br>• Vagina n= 3<br>• Endometrium n= 3<br>• Gynaecological sarcoma n = 3<br><br>*Treatment*- NR	From gastrostomy placement	Median 57 dasy (range 4-472)	In 49/64 (76.5%) symptomatic well-being was obtained after a few days of PEG	NR

Cunningham 1995<br>Retrospective cohort study<br>USA	To evaluate the use of percutaneous decompression gastrostomy in patients with gynaecologic malignancies. Evaluated for successful gastric decompression, acute and long-term complications, and palliation of symptoms.	20<br><br>Mean 52 (range 31-73)<br>*F* n= 20<br><br>100%	*Diagnosis*<br><br>• Ovary n= 10 patients<br>• Uterine corpus n= 6<br>• Cervix n= 3<br>• Peritoneum n= 1<br>All patients had recurrent gynaecologic malignancies.<br><br>*Treatment*<br>• Three patients had undergone previous paracentesis.<br>• Nineteen patients had undergone at least one prior laparotomy (mean 2.2, range 1–6).<br>• Eight patients had received prior radiation therapy including whole abdominal radiation in 2 patients, extended field radiation in 4 patients, and pelvic radiation in 2 patients.	Length of tube placement	Mean 53 days (range 7-184 days) (Seventeen patients continued gastrostomy drainage until the time of death.)*	All patients had significant relief of nausea and emesis except two who had persistent nausea despite adequate gastric decompression	NR
Diver 2013<br>Retrospective cohort study<br>USA	To review a single institution's experience with gastrostomy tubes (GTs) performed for malignant bowel obstruction from gynecologic cancers.	115<br><br>Median- 57 (range 26–88)<br>*F* n = 115<br><br>100%	*Diagnosis*<br><br>Ovarian/fallopian tube/PPC n= 96<br><br>Cervical n= 6<br>Uterine (epithelial and stromal) n =13<br><br>*Treatment*<br><br>Chemotherapy (No. of lines of received prior to GT)<br>1 n= 20<br>2 n= 22	From gastrostomy placement	Median 5.57 weeks (range 1 day–5.5 years)	NR	NR

			3 or more n= 67<br>• Unknown- 6				
Dittrich<br>2017<br>Retrospective cohort study<br>Germany	Investigate the quantity of symptom relief realized with PEG and the corresponding complications.	76<br><br>Median- 66 (range 23-86)<br>F n= 53<br>M n =22<br><br>100%	*Diagnosis*<br><br>•Ovarian n= 24<br>•Colorectal n= 13<br>•Pancreatic n= 12<br>•Small intestine n= 5<br>•Gallbladder/biliary tract n= 5<br>•Gastric n= 4<br>•Breast n= 3<br>•CUP n= 3<br>•Other n= 6<br><br>*Treatment*- NR	From gastrostomy placement	Median 28 days (range 2–440).	Without a NG tube or PEG, the mean frequency of vomiting per day was 2.2. The use of a NG tube led to a reduction of daily vomiting to 0.8, and the PEG to a more decreased value of 0.4. PEG reduced the daily frequency of vomiting to 18% of the initial value and the probability to suffer from nausea to 50% (both p < 0.001).	NR
Gauvin<br>2021<br>Retrospective cohort study<br>USA	To better understand the risks, benefits, and practices associated with the placement and maintenance of palliative G-tubes in	55<br><br>Mean ± SD (range), years, 59.5 ±	*Diagnosis*<br><br>• GI, pancreas, or liver n= 24<br>• Thoracic/eosphageal n= 3<br>• Gynaecologic/Genitourinary n= 26<br>• Other n= 2	from the date of gastrostomy placement to the date of death or last follow-	Survivial %<br>30 d 54.8<br>1 y 11.4<br>3 y 9.5	NR	NR

	patients with cancer at our institution	11.3 (35–88)<br><br>100%	*Treatment*<br><br>Chemotherapy within 3 months of placement n= 16	up visit, with patients alive at last follow-up considered censored			
Goldberg 2021 Retrospective cohort study USA	To describe the clinical outcomes after dPEG in patients with MBO and explore patients'understanding of their illness and expectations forthe future	125<br><br>Median 62 years (range, 33-95 years)<br>*F* n= 65<br>*M* n= 60<br><br>100%	*Diagnosis*<br><br>• Colorectal adenocarcinoma n=41<br>• Pancreas/ampullary adenocarcinoma n=30<br>• Gastric/esophageal/GE junction adenocarcinoma n=22<br>• Appendiceal adenocarcinoma n=15<br>• Bile duct/gallbladder adenocarcinoma n=8<br>• Small intestine adenocarcinoma n=4<br>• Adenocarcinoma of unknown primary n=2<br>• Colorectal neuroendocrine n=2<br>• Pancreas/ampullary neuroendocrine n=1<br><br>*Treatment*- NR	From gastrostomy placement	Median 37 days (95% CI, 29 to 45 days)	NR	NR
Herman 1992 Retrospective cohort study USA	Report the use of Percutaneous endoscopic gastrostomies for decompression of the	50<br><br>Mean- 54 (range 20 to 79)	*Diagnosis*<br><br>•Ovary- 26<br>•Colon- 5<br>•Stomach- 5	Length of tube placement	Mean 66 days (range, 8 to 639 days)	Only three patients (6%) continued to have recurrent nausea post-	NR

	obstructed gastrointestinal tract.	*F* n = 42<br>*M* n= 11<br><br>100%	•Pancreas- 4<br>•Melanoma- 4<br>•Endometrial- 4<br>•Breast- 1<br>•Renal- 1<br><br>*Treatment*<br><br>Non-surgical candidates and had failed chemotherapy			procedure that was not due to drainage tube impaction and was felt to be a result of central nervous system alteration. Following successful placement, 87% of the patients tolerated a full liquid diet and 56% were also able to ingest soft foods.	
Issaka<br>2014<br>Retrospective cohort study<br>USA	To determine the outcomes of VPEG placement in patients with advanced malignancy	96<br><br>median 57 (range 21–90)<br>F n= 57<br>M n= 39<br><br>100%	*Diagnosis*<br><br>•Colorectal n= 26<br>•Pancreas n= 18<br>•Gynaecological n= 17<br>•Gastric n= 6<br>•Other n= 29<br><br>*Treatment-* NR	From gastrostomy placement	mean 135 ± 347.9 days (range 5–2,772 days)	Complete relief of nausea and vomiting was observed in the majority of patients (n = 81, 91.0 %)	NR
Jolicoeur<br>2003<br>Retrospective cohort study<br>Canada	To explore whether or not successful symptom control was achieved when using a PEG tube in patients with recurrent ovarian/peritoneal	24<br><br>Age- NR<br>F n= 24<br><br>100%	*Diagnosis*<br><br>Ovarian n= 24<br><br>*88% (n=21) also presented with a diagnosis of recurrent/progressive ovarian cancer	From gastrostomy placement	median 42 days (range 5 to 1226)	At the time of discharge, 75% of patients were relieved of nausea and 88% no longer	NR

	cancer and bowel obstruction		*Treatment*<br><br>19 patients had been surgically debulked and 22 had received chemotherapy			vomited; 17% of patients complained of abdominal cramping and abdominal bloating was experienced by only 17% of patients. By discharge, 92% of patients were able to resume some type of oral intake.	
Kawata 2014 Retrospective cohort study Japan	To evaluate the outcomes and safety of PEG for bowel decompression in a relatively larger number of patients with malignant bowel obstruction	76<br><br>Median 62 years (range 21–83)<br>*F* n=- 32<br>*M* n= 44	*Diagnosis*<br><br>•Pancreatic cancer n= 27<br>•Colorectal cancer n= 9<br>•Gastric cancer n= 8<br>•Duodenal cancer n= 2<br>•Other gastrointestinal cancer n= 9<br>•Gynecological cancer n=7<br>•Urological cancer n=5<br>•Other primary malignancy n= 9<br><br>Peritoneal carcinomatosis - Absent 20, Present 56.<br><br>*Treatment*<br><br>•Chemotherapy n= 46<br>•Best supportive care n= 30	From gastrostomy placement	median 63 days (range 8–444)	Successful symptom relief was achieved in 53/55 of our patients	NR

Lilley 2018 Retrospective cohort study USA	To compare the following outcomes after treatment for MBO among patients with stage IV ovarian or pancreatic cancer: 1) survival; 2) readmission for MBO; 3) EOL care outcomes, including hospice enrollment, ICU care in the last days of life, and location of death in an acute care hospital.	249<br><br>65-74 years n= 119 (47.8%)<br>75-84 years n= 109 (43.8%)<br>≥ 85 years n = 21 (8.8%)<br>*F* n= 212<br>*M* n= 37	*Diagnosis*<br><br>Ovarian n= 181<br>Pancreas n= 68<br><br>*Treatment*- NR	From admission with MBO	Median 38 (IQ range, 23-69) days.	NR	NR
Merchant 2020 Retrospective cohort study Canada	To (1) examine the incidence of IO, (2) describe current management of IO, and (3) explore the relationship between IO management and patient outcomes in a population-based cohort of patients with colorectal, ovarian, gastric, and pancreatic cancers in Ontario, Canada, in the last year of life.	202<br><br>Age- NR<br><br>Sex- NR<br><br>100%		Not defined	Median survival 47 days (IQR: 27-78)	NR	NR
Pothuri 2005 Retrospective cohort study	To analyze the feasibility of using percutaneous endoscopic	94<br><br>Age- Mean 56	*Diagnosis*<br><br>Ovarian n=	From gastrostomy placement	median weeks (95% CI, 6–10)	Symptomatic relief—the absence of nausea or	NR

USA	gastrostomy (PEG) tube placement in ovarian cancer patients with malignant bowel obstruction and to analyze the outcome of these patient	years (range 27-78)<br>F n= 94<br><br>100%	The majority of the patients (97%) had stage III or IV disease.<br><br>*Treatment*<br><br>89% had received three or more chemotherapy regimens prior to PEG tube placement.<br>Mean laparotomies prior to PEG tube placement was 1.94 (range, 1–6).<br>Thirty-seven of 94 patients had previous gastrointestinal surgery.			vomiting—was noted in 86 (91%) of 94 patients with successful PEG tube placement, the mean number of days to achieve relief was 1.7 days. Diet tolerated with and without the PEG tube being clamped was as follows: none, 3; sips, 9; liquids, 40; soft/regular, 40; and unknown, 2.	
Rath<br>2013<br>Retrospective cohort study<br>USA	To evaluate perioperative and survival outcomes of ovarian cancer patients undergoing percutaneous upper gastrointestinal decompression for malignant bowel obstruction	53<br><br>Age-median 60 years (range 38–78 years)<br>F n= 53<br><br>100%	*Diagnosis*<br><br>•Ovarian<br>•Fallopian tube<br>•Primary peritoneal cancer<br>*Numbers not reported<br><br>*Treatment*<br><br>Chemotherapy- median of 3 regimens, median time since last cycle of chemotherapy prior to	From gastrostomy placement	median s 46 days (range 2–736 days)	Forty-nine patients (92.5%) experienced control of symptoms (nausea and vomiting), defined as resolution of symptoms prior to	NR

			gastrostomy placement was 1.4 months.			discharge; however, 46 patients required supplemental anti-emetic medication. Forty-eight (91%) patients were able to tolerate some form of oral intake: regular diet (8), soft diet (6) and liquid diet (34). Two patients tolerated tube feeds only and 2 patients were unable to tolerate any form of dietary intake.	
Scheidbach 1999 Retrospective cohort study Germany	In addition to establishing indications and outcome, were to identify specific aspects of tube placement and to determine the incidence of complications.	24<br><br>Mean age 64 years (range 37 – 83 years)<br><br>Sex – NR	NR	From gastrostomy placement	Average survival for the patients discharged home (20/24) was 21 weeks (range, 6–52 weeks)	documented as 24/24 (100%) relief as NGT not needed. Twenty-two patients (92%) were also soon able to take liquids or soft	NR

		100%				foods by mouth.	
Teriaky 2012 Retrospective cohort study Canada	To determine the efficacy of venting PEG tubes in relieving nausea and vomiting	7<br><br>Age- mean 62 (range 37-82)<br>F n= 4<br>M n= 3<br><br>100%	*Diagnosis*<br><br>•Colon n= 3<br>•Pancreas n = 2<br>•Other n= 2<br>*Treatment*<br><br>•Surgery n= 6<br>•Adjuvant chemotherapy n= 5<br>•Radiotherapy n= 2<br><br>None of the patients underwent any further surgery, chemotherapy, or radiation therapy after insertion of a venting PEG.	From gastrostomy placement	mean a 119 days (range 6-484 days)	There was relief of nausea and vomiting in 6 (86%) patients on the first day after PEG tube insertion, which persisted throughout hospitalization. Two patients were able to tolerate liquids and 3 patients were able to tolerate food.	NR
Vashi 2012 Retrospective cohort study USA	Not stated	73<br><br>Age- Mean 53.3 years (range: 22.3–69 years)<br>F n= 54<br>M n= 19<br><br>100%	*Diagnosis*<br><br>Majority (n = 27) had cancers of the female genital tract and stage III (n = 22) or IV (n = 27) disease at diagnosis, others not reported.<br>All had advanced abdominal carcinomatosis-induced bowel obstruction.<br><br>*Treatment*- NR	From gastrostomy placement	"average" 83.7 days (range: 20–338 days)	All patients had the PEG tube functioning well after the procedure with immediate relief of obstructive symptoms of persistent nausea and vomiting.	NR

Zucchi 2016 Retrospective cohort study Italy	The aim of this study is to examine, in a large single-centre cohort of 158 successive patients with MBO and abdominal carcinomatosis from advanced gynaecological and gastroenteric cancer, the efficacy and outcomes of PEG.	158<br><br>Age- NR<br><br>Sex- NR<br><br>100%	*Diagnosis*<br><br>• Colon carcinoma n= 13<br>• Gastric carcinoma n= 7<br>• Gallbladder carcinoma n= 2<br>• Breast carcinoma n= 2<br>• Pancreas carcinoma n= 2<br>• Ovarian carcinoma n= 96<br>• Portio carcinoma n= 6<br>• Endometrial carcinoma n= 8<br>• Uterine sarcoma n= 6<br><br>Malignant small bowel obstruction from abdominal-pelvic carcinomatosis<br><br>*Treatment*<br><br>All patients had at least one previous gastrointestinal surgical procedure (19.7 % had one surgical procedure, 42.2 % had two, 28.1 % had three, 7.7 % had four, 2.1 % had five)	From gastrostomy placement	Median 57 days (Range 4-472)	110 (77.4 %) experienced complete relief from nausea and vomiting and were able to resume oral liquids and small amounts of soft food intake for a median of 57 days with self-reported satisfaction. Twelve patients (8.4 %) had only nausea, while 20 (14 %) had persistent vomiting.	25 patients had an SDS score properly evaluated. Sixteen (64 %) improved (41 vs 32.6, pre- and post-PEG median scores, respectively, p < 0.01), two (8 %) at a further assessment showed the same scores as at baseline, and seven (28 %) had non-significant worsening (30.85 vs 36.14, p=0.18) of QoL. Of the 16 patients who showed an improvement in the QoL, nine reported an improvement of symptoms

							at physical (19.6 vs 14.75, $p < 0.01$), psychological (10.1 vs 7.3, $p < 0.05$), and somatopsychic levels (11.25 vs 9.2, $p < 0.05$). Regarding diet tolerance, all were able to resume oral liquid and small amounts of soft food intake. Of the remaining seven patients, one reported improvement at the physical level, three at the psychological level, and three at the somatopsychic level. The worsening of global QoL was determined by

							the persistence of the physical symptoms (14.57 vs 20, $p < 0.05$) while psychological and somatopsychic levels remained stable. Symptom Distress Scale (SDS) of McCorkle and Young (1978)
