# Supplementary material for: Inoperable malignant bowel obstruction: palliative interventions outcomes – mixed-methods systematic review
Source: BMJ Support Palliat Care. 2022 Jul 19;13(e3):e515–27. doi: 10.1136/bmjspcare-2021-003492 (PMC10850628; doi:10.1136/bmjspcare-2021-003492)
Supplement: Supplementary data [file bmjspcare-2021-003492supp004.pdf]

Health Service Utilisation and Place of Death

<table>
<tr><th>Study</th><th>Discharge status</th><th colspan="3">Place of death</th><th>Length of stay</th><th>Readmissions/ hospitalisations</th><th>ICU admissions</th></tr>
<tr><td colspan="8">Parenteral Nutrition</td></tr>
<tr><td>August 1991</td><td></td><td colspan="3"></td><td></td><td>Three patients readmitted to evaluate complications possibly related to HPN.</td><td></td></tr>
<tr><td>Bond 2019</td><td></td><td colspan="3"></td><td>Follow a series of improvement measure length of stay was reduced from 29.4 days to 10.1 days</td><td></td><td></td></tr>
<tr><td rowspan="4">Brard 2006</td><td rowspan="4"></td><td></td><td>TPN n= 28</td><td>No TPN N = 27</td><td rowspan="4"></td><td rowspan="4"></td><td rowspan="4"></td></tr>
<tr><td>Home</td><td>11 (39%)</td><td>10 (37%)</td></tr>
<tr><td>Hospital</td><td>9 (32%)</td><td>7 (26%)</td></tr>
<tr><td>Hospice</td><td>8 (29%)</td><td>10 (37%)</td></tr>
<tr><td>Chouhan 2016</td><td></td><td colspan="3"></td><td></td><td>Sixty-three (76.8%) patients required hospitalization after the initiation of systemic chemotherapy and TPN. The median number of hospitalizations was 2 (range, 1–11), and the median time spent hospitalized was 26.5 days (range, 4–167 days). On average, patients spent 38% (range, 1–100%) of their remaining lives in the hospital.</td><td>23 of those hospitalised patients admitted to the intensive care unit.</td></tr>
<tr><td>Duerksen 2004</td><td>Five of nine were discharged home.</td><td colspan="3"></td><td></td><td>One patient had five admissions.</td><td></td></tr>
</table>

Dzierianowski 2021				Median 0 (range 0-13)	
Keane 2018		Most patients passed away in their homes or hospice (77.9%)			
King 1993				124 hospitalisations occurred during HPN. Thirty-seven admissions were for cancer therapy, 76 were for disease complications, and 11 were for HPN-related complications. Mean number of admissions was 2.0, median was 1, with a range of 0-11. Twenty-six percent of HPN patients had no admissions.	
Patel 2021			Median length of stay was 13 days, with greater lenth of stay in those referred for PN than those who were not (28 vs. 9 day, P= 0.0001)	The median number of readmissions was 1 (range 1-12).	Median number of ICU admission was 0
Palliative Venting Gastrostomy					
Adelson 1993		1 patient died in hospital on supportive care.			
Brooksbank 2002	Following insertion of the gastrostomy, 20 patients could be discharged home. Four other patients were transferred to country hospitals to	Six patients died at home and two patients were readmitted for the final few hours of life.			

	continue care nearer to home.				
Cannizarro 1995		Four in hospital			
Cunningham 1995	Twelve (60%) patients returned home for terminal care for 3 to 173 days (mean 70)				
Dittrich 2017	44 patients (59%) were discharged (home 36 patients, inpatient hospice 5, nursing facility 3)	Hospital n= 46<br>Home n= 23<br>Inpatient hospice n= 6	Median length of stay was 8.5 days (range 1–28)	Readmission rate was 48% (21/44 patients, range 1–5 readmissions	
Diver 2013				48 women (42%) were readmitted to the hospital at least once after GT placement due to GT related events	
Gauvin 2021	•Home n= 22<br>•Rehabilitation/SNF n= 7<br>•Hospice n= 25				
Goldberg 2021	Hospice enrolment<br>Yes n= 95<br>No n= 22	Discharge status<br>Alive n= 117<br>Dead n= 8	Length of stay for those discharged alive, days, median 10.0 (range 0.0-38.0)		
Issaka 2014				Four patients were re-admitted for recurrent symptoms due to VPEG tube dysfunction caused by clogging, and all were successfully replaced. Seven patients (7.9 %) were re-admitted for obstructive	

				symptoms despite a functioning VPEG tube.	
Jolicouer 2003	Fifty-eight per cent were discharged home with a primary caregiver. A quarter of patients were discharged to a hospice. Thirteen per cent died in hospital within 26 days of being admitted for their bowel obstruction and one patient was transferred to a community hospital close to home.	Thirteen per cent died in hospital		Of the 20 patients discharged from the acute care setting, 30% (n=6) required re-admission post-PEG insertion. A total of 20 readmissions were required, 70% did not require re-admission. The first re-admission occurred on average within 21.7 days of the patient being discharged (range from five to 60 days).	
Lilley 2018	Hospice enrolled n = 190 (76.4%)	Death in hospital n= 34 (13.8%)			ICU Care n= 32 (12.8%)
Merchant 2020		Death in hospital- 67/135	Median length of stay 23 days (IQR: 13-37)	Patient readmissions for MBO- 16/135 (11.9%)	
Pothuri 2005		Seventy-five (85%) of 88 patients died at home or under hospice care.	Five patients were able to leave the hospital the day of PEG tube placement, and 88 remained in the hospital for a mean stay of 6.9 days after the procedure.		

Rath 2013	71.7% were discharged home; 9.4% to an inpatient hospice facility, 15.1% to a nursing facility, and there were two inpatient deaths. Sixteen patients (30.2%) opted for hospice services	Two inpatient deaths	The median length of stay prior to placement of PDT was 6 days (range 1–27).		
Scheidbach 1999	83% discharged to home after PEG/PEJ (not stated where they died), 4 died in hospital.	Four hospital deaths	Average stay after PEG/PEJ placement was 6 days, range 3 - 31 days - except for 4 patients who could not be discharged secondary to disability - all died in hospital within days (up to 4 weeks) after treatment.		
Teriaky 2012		3 at home, 4 in hospital (3 of which after re-admission)		4 (67%) patients requiring re-admission for end stage palliation in hospital. The average length of time spent at home prior to readmission for in-hospital palliation was 126 days (range 7-467 days)	
Zucchi 2016	Out of 142 patients, 116 (81.6 %) were discharged	Twenty-six (18.3 %) out of 142 deaths occurred during the hospital stay because of disease progression	The median postoperative hospital stay		

			was 9 days (range 3–60).		
