# Supplementary material for: Inoperable malignant bowel obstruction: palliative interventions outcomes – mixed-methods systematic review
Source: BMJ Support Palliat Care. 2022 Jul 19;13(e3):e515–27. doi: 10.1136/bmjspcare-2021-003492 (PMC10850628; doi:10.1136/bmjspcare-2021-003492)
Supplement: Supplementary data [file bmjspcare-2021-003492supp005.pdf]

Qualitative synthesis themes

**Theme**	**Sub-theme**	**Codes**	**Quotations**
Theme one			
A stark decision- Do or Die	No alterative<br><br>Decision has been made	Life or death<br>Take it or leave it<br>Perception of decision making<br>Lifeline	"If I don't have the [HPN], I won't be here. There's no other way, is there?" (69)<br><br>It's either die with food or [HPN] for the rest of your days and I'd sooner live and be on [HPN]" (69)<br><br>No, why would I do that?" Asked about stopping HPN (69)<br><br>"when I get to the point where I've got to say, oh, enough is enough, it won't matter then, but until that point comes then I just have to fight, keep going" (69)<br><br>"there was no choice really, it was one of those take it or leave it, they didn't say that, but it's a take it or leave it, isn't it?" (73)<br><br>"Well, to me it was a no option thing. I don't think they could have done anything else, bar starve me . . . if that's what's keeping me alive, it's what I have to have isn't it. So I don't think [there was] a decision as such, if there was no other . . . if I can't eat, it will be next best thing" (73)<br><br>"Hobson's choice" (73)

			“Certainly yes, I mean what’s the alternative…you just have to go with what the doctors recommend, I think. (75)
Theme two			
Realities of the intervention.	Benefits	Improved quantity and quality of life<br>Valuing activities of daily living<br>Increased energy/strength<br>Symptoms	“spending time with family when you get to, like, my stage, is the most important for everybody” (69)<br><br>“it’s given me, yes, more energy” (69)<br><br>“I think I’ve put a little bit of weight on” (69)<br><br>“It’s keeping her alive really. That’s the big advantage.” (Husband). (69)<br><br>“it’s going to help her do what she wants to do” (Husband). (69)<br><br>“I’m looking forward to her being able to come out of hospital and go home and have the [HPN] at home and, sort of, have some sort of normality to life” (Daughter) (69)<br><br>“Well they explained that it would be helpful for the sickness…stopping the sickness, which it did. I was so grateful for that because it was just projectile all the time. (75)<br><br>“I hated that up my nose (NGT) it was so uncomfortable. It hurt me…it was horrible and uncomfortable in my throat, but this (referring to her PVG tube) isn’t uncomfortable. (75)
	Burdens	Hospitalising home<br>Role of carers<br>Complexity of treatment<br>Loss of normality<br>Emotional loss<br>Lack of information	“initially when this was being discussed with us ... I thought it was probably less medical than what it is” (Daughter). (69)<br><br>“It wasn’t as easy as it was made out to be” (69)

		Recommendations from HCP	"It just becomes a way of life really, you know what I mean, this is how your day goes and this is what it is. A nurse comes and takes it off in a morning and then a nurse comes at night and puts it back on" (Belinda). (69)<br><br>"I've tried creeping, 'cause I don't want to wake him up" (Penny).<br>"I'm awake most of the night listening for her, but she tells me not to help her" (Husband). (69)<br><br>"I am physically falling to bits" (Daughter). (69)<br><br>"what you sign on for when you get married" (Husband) (at the end of the second interview, he reported feeling like a "prisoner") (69)<br><br>"It would be wonderful if I could have even 5 h sleep without a break" (Marilyn). (69)<br><br>"It's difficult, yeah, especially going upstairs, be- cause I've not got much energy, so usually my husband ... has to take it for me" (69)<br><br>"when I go in the shower and everything, I can ... take both tubes off, and I'm a different person" (69)<br><br>"a ball and chain" (Husband). (69)<br><br>"I've done things for him. He can do things for me" (69)<br><br>"as for going out and taking a contract on or something. It's just not feasible" (Husband). (69)<br><br>"(are you able to walk up and down the stairs?) …not when carrying my bags (referring to her PVG, PN and syringe pump), but X (partner) carries those either behind or in front of me." (75)

			"My husband has been in a lot of discomfort, it has been leaking all the time, he's being changed numerous times a day, the beds have to be changed and now his skin is all sore." (75)<br><br>"You can smell it though, even if it's not leaking. I feel like…it smells like sewage, it's not faecal, it's worse than that, it's a sewage smell and I feel like I can smell it all the time and anyone who is anywhere near me can smell it. It is making me quite paranoid, I am constantly asking my husband if he can smell it…I don't get embarrassed too easily, but I do find that quite difficult to deal with)" (75)<br><br>when I got down to radiology, Dr X (Consultant IR) came and explained it all to me and I was even more anxious then because I sort of then understood what was happening… (not given any written information pre-PVG)" (75)<br><br>"(Referring to ward nurses): …one nurse who was giving it a clean said, you don't rotate this one…that other nurse, she said something about I'll rotate it and I said oh well I've been told not to rotate mine." (75)
